# Topology-driven negative sampling enhances generalizability in protein–protein interaction prediction

**DOI:** 10.1093/bioinformatics/btaf148

**Published:** 2025-04-07

**Authors:** Ayan Chatterjee, Babak Ravandi, Parham Haddadi, Naomi H Philip, Mario Abdelmessih, William R Mowrey, Piero Ricchiuto, Yupu Liang, Wei Ding, Juan Carlos Mobarec, Tina Eliassi-Rad

**Affiliations:** BioClarity AI, Boston, MA 02130, United States; Bioinformatics and Data Science, Alexion AstraZeneca Rare Disease, Boston, MA 02210, United States; Network Science Institute, Northeastern University, Boston, MA 02115, United States; Bioinformatics and Data Science, Alexion AstraZeneca Rare Disease, Boston, MA 02210, United States; Network Science Institute, Northeastern University, Boston, MA 02115, United States; Department of Physics, Northeastern University, Boston, MA 02115, United States; Bioinformatics and Data Science, Alexion AstraZeneca Rare Disease, Boston, MA 02210, United States; Bioinformatics and Data Science, Alexion AstraZeneca Rare Disease, Boston, MA 02210, United States; Bioinformatics and Data Science, Alexion AstraZeneca Rare Disease, Boston, MA 02210, United States; Bioinformatics and Data Science, Alexion AstraZeneca Rare Disease, Boston, MA 02210, United States; Bioinformatics and Data Science, Alexion AstraZeneca Rare Disease, Boston, MA 02210, United States; Bioinformatics and Data Science, Alexion AstraZeneca Rare Disease, Boston, MA 02210, United States; Bioinformatics and Data Science, Alexion AstraZeneca Rare Disease, Boston, MA 02210, United States; Protein Structure and Biophysics, Discovery Sciences, R&D, AstraZeneca, Cambridge, UK; Network Science Institute, Northeastern University, Boston, MA 02115, United States; Khoury College of Computer Sciences, Northeastern University, Boston, MA CB2 0AA, United States; Santa Fe Institute, Santa Fe, NM 87501, United States

## Abstract

**Motivation:**

Unraveling the human interactome to uncover disease-specific patterns and discover drug targets hinges on accurate protein–protein interaction (PPI) predictions. However, challenges persist in machine learning (ML) models due to a scarcity of quality hard negative samples, shortcut learning, and limited generalizability to novel proteins.

**Results:**

In this study, we introduce a novel approach for strategic sampling of protein–protein noninteractions (PPNIs) by leveraging higher-order network characteristics that capture the inherent complementarity-driven mechanisms of PPIs. Next, we introduce Unsupervised Pre-training of Node Attributes tuned for PPI (UPNA-PPI), a high throughput sequence-to-function ML pipeline, integrating unsupervised pre-training in protein representation learning with Topological PPNI (TPPNI) samples, capable of efficiently screening billions of interactions. By using our TPPNI in training the UPNA-PPI model, we improve PPI prediction generalizability and interpretability, particularly in identifying potential binding sites locations on amino acid sequences, strengthening the prioritization of screening assays and facilitating the transferability of ML predictions across protein families and homodimers. UPNA-PPI establishes the foundation for a fundamental negative sampling methodology in graph machine learning by integrating insights from network topology.

**Availability and implementation:**

Code and UPNA-PPI predictions are freely available at https://github.com/alxndgb/UPNA-PPI.

## 1 Introduction

Proteins play a central role in essential biological processes, including catalyzing reactions, transporting molecules, responding to pathogens in the immune system, and facilitating cell-to-cell signal transduction (Rao *et al.* 2014, Das *et al.* 2023). Moreover, crucial cellular processes vital for our health, such as DNA replication, transcription, translation, and transmembrane signal transduction, depend on specific functional proteins (Lu *et al.* 2020). These fundamental biological activities are regulated through protein complexes, typically governed by protein–protein interactions (PPIs) (Rual *et al.* 2005, Stelzl *et al.* 2005). In humans, deviations from typical patterns of PPIs and protein complexes can either cause or indicate a disease state (Kuzmanov and Emili 2013). Numerous computational methods have been developed to uncover the etiology of diseases form PPIs (Kann 2007). However, the incompleteness of the human interactome (Vidal 2016, Dimitrakopoulos *et al.* 2022) hinders the understanding of pathogenic and physiological mechanisms that trigger the onset and progression of diseases (Safari-Alighiarloo *et al.* 2014), and hence in the development of novel therapeutic strategies (Jaeger and Aloy 2012).

**Figure 1.**
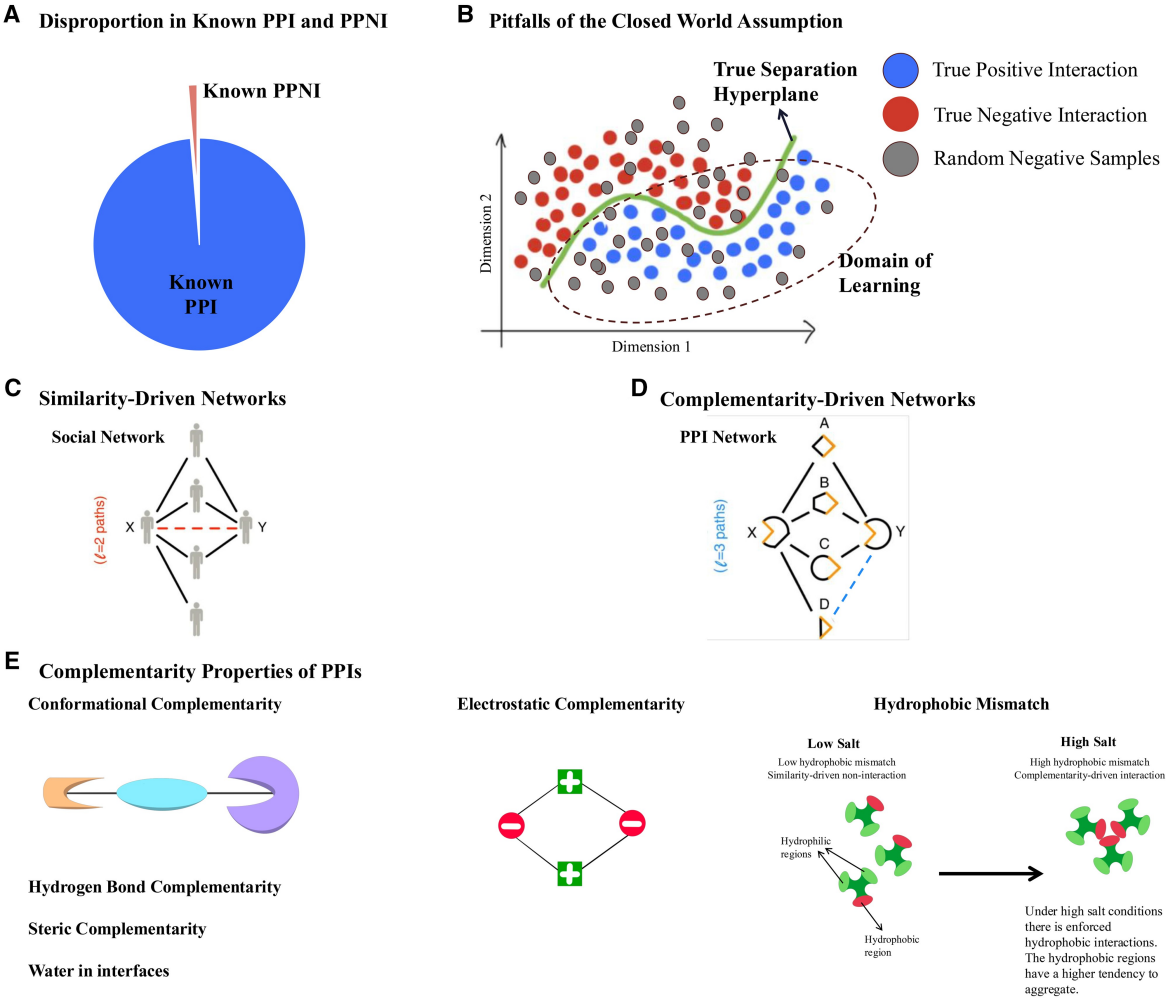
Status quo in machine learning-based PPI prediction. (A) We observe a substantially higher number of PPI samples in the existing databases compared to PPNI samples. The lack of true negative samples is a major obstacle in training reliable machine learning models for PPI prediction. (B) Traditional machine learning training methodology utilizes random sampling from the complement graph ($G^{c}$) of the training PPI network (*G*) to obtain negative examples. However, this approach violates the closed-world assumption, i.e. not observing a PPI does not imply noninteraction between those proteins. Hence, the traditional approach fails to create high-quality PPNI and shifts the domain of learning in the loss function manifold, hampering the machine learning models from learning the true separation hyperplane between PPIs and true PPNIs. (C and D) In similarity-driven networks, entities that are alike are connected by links. For example, in social networks, people with similar interests are connected. On the other hand, in complementarity-driven networks, entities with opposing properties are linked (courtesy of Kovács *et al*. 2019). Nodes *x* and *y* are predicted to be connected based on l=2 and l=3 paths in (C) and (D), respectively. (E) We list six complementary properties of proteins that are major contributors to protein–protein interactions (Veselovsky *et al*. 2002). Leveraging the complementary nature of PPI networks has recently gained much attention and bifurcates the machine learning approaches for PPI prediction from those widely used in social network analysis.

Experimental PPI databases such as BioPlex (Huttlin *et al.* 2015), STRING (Szklarczyk *et al.* 2021), APID (Alonso-López *et al.* 2019), BioGRID (Stark *et al.* 2006), CoFrac (O’Reilly *et al.* 2023), CORUM (Giurgiu *et al.* 2018), HuRI (Luck *et al.* 2020), HINT (Das and Yu 2012), and HIPPIE (Alanis-Lobato *et al.* 2017), to name a few, capture human PPIs observed via Affinity Purification—Mass Spectrometry (AP-MS) (Dunham *et al.* 2012) and Yeast-to-hybrid (Y2H) assays (Coates and Hall 2003). However, none of the PPI databases report the failed experiments, i.e. the noninteractions, creating a scarcity of high-quality protein–protein noninteractions a.k.a. hard negatives (Robinson *et al.* 2021) in training ML models (see Fig. 1A). Negatome (Blohm *et al.* 2013) stands out as the sole database that has endeavored to tackle this concern; however, it encompasses merely 2424 interactions linked to human proteins (Consortium 2020) and is limited by specific environmental constraints. As a result, it falls short of capturing the essential interaction mechanisms required for training machine learning models to prioritize large-scale screening. Zhao *et al.* (2022) provided triple-layer validated (Srivastava *et al.* 2016) negatives, yet only 58 pairs are provided for the human proteins reported in UniProt (Consortium 2020). Furthermore, these databases suffer from high rates of false negatives and false positives (Xia *et al.* 2010a,b, You *et al.* 2010), which, combined with the limitations of the traditional ML-based negative sampling methods, significantly bias the PPI predictions (Ben-Hur and Noble 2006). For example, some authors propose generating high-quality noninteractions by considering pairs of proteins with distinct cellular localization (Thul *et al.* 2017), presumably hindering their participation in biologically relevant interactions (Jansen *et al.* 2003, Jansen and Gerstein 2004). This method samples protein pairs as negative samples from the compartment pairs that do not share any protein (see Supplementary Fig. S1). Alternatively, other authors adopt a simpler approach, randomly selecting protein–protein noninteraction (PPNI) pairs from the entire set of protein pairs that are not known to interact (Gomez *et al.* 2003, Qi *et al.* 2004, Zhang *et al.* 2004, Ben-Hur and Noble 2005). However, both negative sampling scenarios produce a skewed distribution of negative examples, resulting in overly optimistic estimates of classifier accuracy (Jansen *et al.* 2003, Wang *et al.* 2004, Martin *et al.* 2005). Furthermore, Bardes *et al.* (2022) have shown that the necessity of negative sampling while training semi-supervised models for predicting similarity between two entities can be overcome by regularization. Nevertheless, as the similarity of proteins does not necessarily indicate interaction, the need for negative sampling persists in PPI prediction. The complementary nature of the PPI network (Budel and Kitsak 2023) demands hard noninteraction samples for unbiased prediction and improved generalizability. In a recent paper, Li *et al.* (2023) have proposed a heuristic-based hard-negative generation method for link prediction. However, this method lacks biological and chemical rationality in creating protein–protein noninteractions and impedes the interpretability of the predictions. Finally, the complex interdependence of the in-vitro screening experiments on the environmental factors makes it difficult to obtain hard negatives solely driven by the molecular properties of the proteins (Peng *et al.* 2017). In another recent paper, diffusion models have been used for deriving quality negative samples for link predictions (Nguyen and Fang 2024). Understanding the evolutionary reasoning for protein interactions and noninteractions is a complex task. However, recent research has unveiled the complementarity-driven mechanisms (Budel and Kitsak 2023) driving PPIs, which include an electrostatic charge or Coulombian complementarity (Grassmann *et al.* 2023), hydrophobic mismatch (Jernigan *et al.* 2022), conformational complementarity (Chen *et al.* 2020b), hydrogen bond complementarity (Veselovsky *et al.* 2002), etc. (see Fig. 1C). Therefore, delineating interactions and noninteractions based on the complementary nature of the PPI network not only facilitates the generation of hard negative samples for training PPI prediction models but also enhances the mechanistic interpretability of these predictions.

Historically, the scarcity of hard negatives and the pronounced imbalance between edges and nonedges during the training of machine learning models for link prediction tasks have led to the practice of obtaining negative samples through random sampling from the complementary graph (Yang *et al.* 2020). Nevertheless, it is noteworthy that random negative samples can introduce substantial bias into the separation hyperplane learned by the classifier, as illustrated in Fig. 1B. Selecting an appropriate set of hard negatives is essential for acquiring the relevant hyperplane and enhancing generalizability in machine learning (Robinson *et al.* 2021).

On a separate note, observation bias (Chatterjee *et al.* 2023a,b) and shortcut learning (Geirhos *et al.* 2020, Chatterjee *et al.* 2023c) are prevailing in the ML-based PPI prediction models due to selection and laboratory biases in the PPI databases (Gillis *et al.* 2014). The excellent transductive (see Supplementary Section S2, Supplementary Figs S2 and S3) cross-validation performances of the majority of the state-of-the-art (SOTA) PPI prediction models (Hu *et al.* 2021a, Jha *et al.* 2022), falsely exaggerated by topological shortcuts (Bonner *et al.* 2022, Chatterjee *et al.* 2023a,c), are often misleading and not biologically interpretable. Multiple recent papers have focused on the poor performance of state-of-the-art link prediction models on low-degree nodes (Chatterjee *et al.* 2023b, Ju *et al.* 2023, Li *et al.* 2023). GraphPatcher (Ju *et al.* 2023) proposes a novel test-time augmentation method to improve link prediction performance for the low-degree nodes. However, the approach is infeasible for unseen nodes, making the prediction task for never-before-seen nodes of greater difficulty (Chatterjee *et al.* 2023b), which maps to the scenario of predicting interactions between novel proteins. Therefore, inductive tests have garnered significant attention recently for their capability to reveal the authentic predictive efficacy of machine learning models (see Supplementary Fig. S2). This allows for the assessment of a model’s performance on novel entities, ultimately contributing to enhanced generalizability and interoperability (Szymborski and Emad 2022, Chatterjee *et al.* 2023c). Recent studies (Szymborski and Emad 2022, Albu *et al.* 2023) emphasize the importance of going beyond the PPI network topology in machine learning and advocate for the incorporation of inductive tests in constructing models with biological utility and interpretability. Yet, intricate calibration of these models via fine-tuning (Kun *et al.* 2021) and embedding regularization limits their generalizability within particular databases and families of proteins (Szymborski and Emad 2022).

Finally, using the appropriate performance metric is essential for evaluating the true performance of an ML model (Lichtnwalter and Chawla 2012, Yang *et al.* 2014). Global performance metrics like precision, recall, the area under the receiver-operating characteristics (AUROC), the area under the precision–recall curve (AUPRC), and accuracy fail to capture the local performance in the PPI network (Dunham and Ganapathiraju 2021). Hence, performance metrics like Hits@TopK (Li *et al.* 2020) and mean reciprocal rank (MRR) (Craswell 2009), which incorporates local evaluation (Dick and Green 2018), are necessary for assessing an ML model for novel, unseen proteins in inductive tests. Furthermore, the evaluation of negative predictions is often overlooked. However, the ability of an ML model to distinguish the positives from the negatives largely determines its predictive power (Vihinen 2012). Yet, a systematic approach is absent in evaluating the PPI prediction models.

The **contributions** of this work are as follows:

1. We introduce UPNA-PPI (Unsupervised Pre-training of Node Attributes tuned for PPI), an ML pipeline that bridges the existing gaps in the sequence-to-function PPI prediction methodology by
  - including a method for obtaining high-quality protein–protein noninteraction (PPNI) pairs, which utilizes the PPI network topology;
  - connecting PPNI pairs to complementary generation mechanisms through hyperbolic embedding space;
  - improving inductive link prediction performance in PPI using topological negatives for training a Two-Shot Learning architecture, i.e. UPNA-PPI; and
  - enabling interpretability and transferability of UPNA-PPI prediction across protein families and homodimers.
2. We propose local performance metrics to evaluate both interaction and noninteraction predictions, quantifying the distinguishing power of any UPNA-PPI prediction model.
3. We predict and validate interactions between understudied and difficult-to-purify G protein-coupled receptors (GPCRs).

## 2 Materials and methods

### 2.1 Merging and consolidating PPI databases

Consolidating the human interactome using the existing experimental databases is challenging (Ramani *et al.* 2005). While the majority of the existing PPI databases often report only the gene identifiers such as gene symbols (Bruford *et al.* 2020), Entrez ID (Maglott *et al.* 2010), Ensembl ID (Martin 2022), etc., newer databases like BioPlex (Huttlin *et al.* 2015) report the protein IDS such as UniProt ID (Consortium 2020), PDB ID (PDB), etc. Ambiguity in gene-to-protein mapping combined with the annotation inconsistency in these databases makes the task of merging the PPI data difficult (Lehne and Schlitt 2009). Furthermore, the majority of the computational tools for exploratory biology, including network science-inspired disease module identification algorithms (Ghiassian *et al.* 2015, Wang and Loscalzo 2018), demand a coarse-grained representation of the interactome. ML-based PPI prediction methods (Hu *et al.* 2021b, Jha *et al.* 2022, Szymborski and Emad 2022) use UniProt IDs to specify proteins, and hence disregard a big portion of available experimental PPI data. For the aforementioned reasons, we coarse-grain human protein IDs from UniProt to gene Entrez IDs. Establishing a one-to-one correspondence between human genes and amino acid sequences involves selecting the longest amino acid sequence from all protein isoforms associated with a given gene. We have merged multiple experimental PPI databases such as BioPlex (Huttlin *et al.* 2015), STRING (Szklarczyk *et al.* 2021), APID (Alonso-López *et al.* 2019), BioGRID (Stark *et al.* 2006), CoFrac (O’Reilly *et al.* 2023), CORUM (Giurgiu *et al.* 2018), HuRI (Luck *et al.* 2020), HINT (Das and Yu 2012), and HIPPIE (Alanis-Lobato *et al.* 2017) to obtain our interaction data.

### 2.2 GoldPPI

To accumulate high-confidence PPI data, we filter the experimental PPI databases for samples with multiple experimental validations. We use three AP-MS databases, which report the number of experiments/publications validating the interaction: APID (Alonso-López *et al.* 2019), HINT (Das and Yu 2012), and HIPPIE (Alanis-Lobato *et al.* 2017). Thereafter, we filter for the PPI samples that have been observed in at least 3 experiments. Thus, we obtain the high confidence PPI, which we use in UPNA-PPI validation and test datasets.

### 2.3 Negatome PPNI

The majority of the PPI databases do not report the noninteractions. Hence, there is a lack of PPNI data in the literature, which has led the majority of the ML approaches to use random negative sampling or subcellular compartmental negatives. We use high-confidence PPNI from Negatome 2.0 (Blohm *et al.* 2013) and NVDT (Zhao *et al.* 2022). These samples have been used in the validation and test of UPNA-PPI.

### 2.4 Step-by-step description of topological negative sampling (TPPNIs)

- Construct the PPI network from experimental interaction data.
- Run the unipartite configuration model on the PPI network to obtain the probabilities for the links between all protein–protein pairs.
- Rank the probabilities of the links in descending order and select the bottom *N* (e.g. *N* = 10 million) protein–protein pairs.
- Compute the number of L3 paths for these bottom N pairs in the original PPI network.
- Select the protein–protein pairs from the bottom N for which the number of induced L3 paths is 0.

### 2.5 Inductive split for training UPNA-PPI

To train UPNA-PPI, we have a total of 706 244 PPIs collected from multiple databases and 3 063 605 PPNIs generated by topological negatives, and collected from Negatome 2.0 (Blohm *et al.* 2013) and NVDT (Zhao *et al.* 2022). Then, following the inductive split methodology from GraIL (Teru *et al.* 2020), we split the proteins into three disjoint groups for training, validation, and testing. The PPIs and PPNIs induced by these proteins are then used for training, validation, and testing, respectively. 2480 PPNIs are from Negatome 2.0 and NVDT, i.e. high confidence and experimentally validated, and are used in validation and test. The remaining PPNIs are generated using our TPPNI method (CL3 filtering), which uses the complementary nature of PPI. All TPPNIs are generated from the experimental human PPIs. Thus, the proposed TPPNIs are a derivative of all known PPIs.

### 2.6 Traditional/simple unipartite configuration model

The Simple Configuration Model (SCM) (Chung and Lu 2002) is an exponential random graph model with the probability of observing a graph configuration $P(G)=\frac{B(G)}{Z}$, where $B(G)=\text{exp}(-\sum_{i=1}^{n}\lambda_{i}d_{i}(G))$, the Lagrange multipliers $\left\{\lambda_{i}\right\}$ are such that $\langle d_{i}\rangle =\sum_{j=1}^{n}p_{ij}=k_{i}$ for all nodes i∈{1,2,…,n}, the Boltzmann factor $B=\sum_{G}B(G)$, $d_{i}(G)=\sum_{j=1}^{n}G_{ij}$ is the degree of the node *i*, and ${\left\{G_{ij}\right\}}_{i,j=1}^{n}$ is the adjacency matrix of the training graph *G*. By entropy maximization, we get the link probability between the nodes *i* and *j* as:


(1) $$p_{ij}=\frac{1}{e^{\lambda_{i}+\lambda_{j}}+1},$$

## 3 Results

### 3.1 Topological PPNI

**Figure 2.**
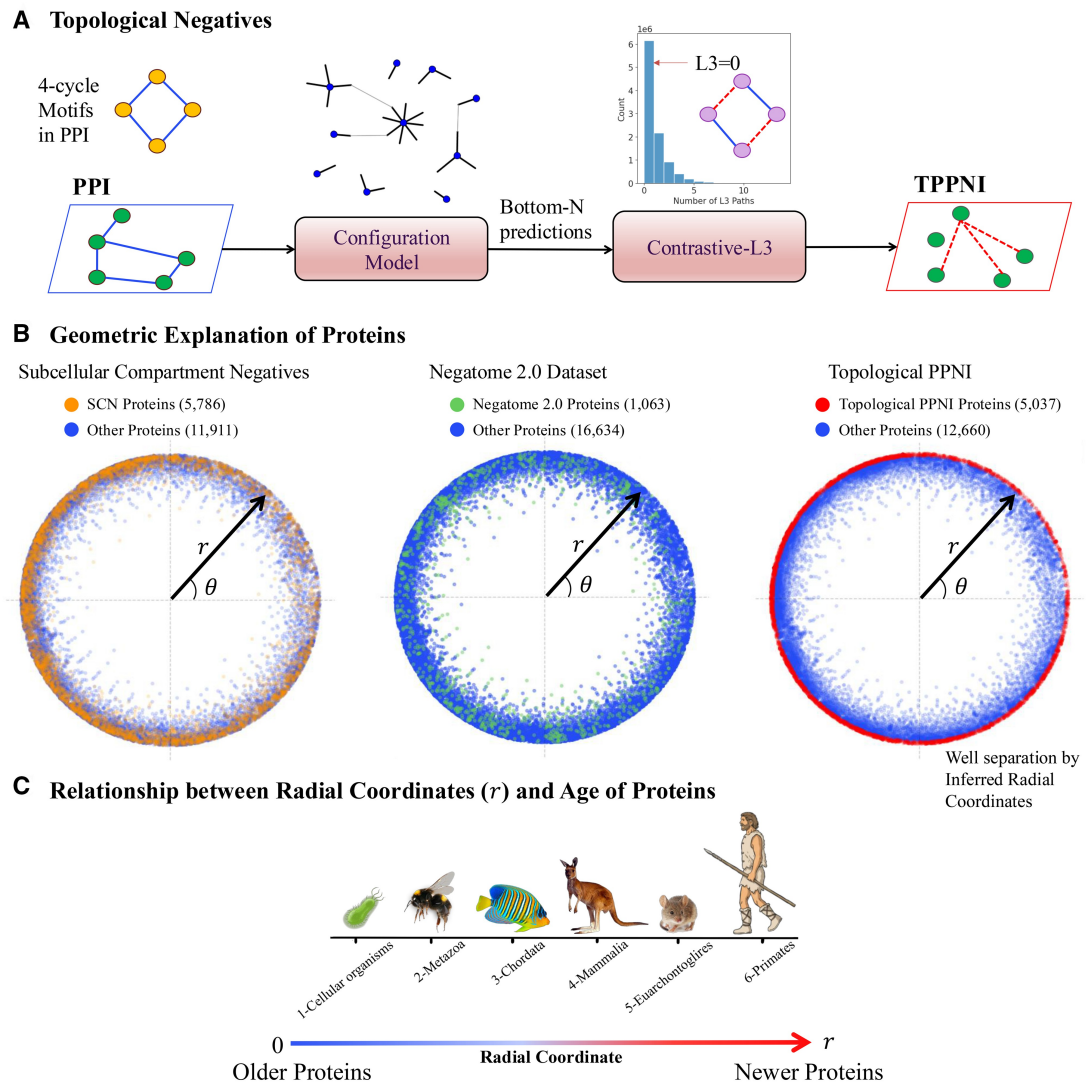
Leveraging PPI topology for PPNI sampling. (A) We propose a novel method for sampling high-quality PPNI leveraging the topology of PPI driven by its complementary nature. First, we run a traditional configuration model to identify the topologically least probable edges via entropy maximization. The bottom-N edges are then used for computing the 4-cycles or L3 paths induced by the protein pairs. (We use *N* = 10 million.) The configuration model helps in reducing the protein-pair search space from 156M down to 10M. Interacting pairs induce many L3 paths in the PPI network. We utilize the inverse hypothesis, namely *Contrastive L3 or CL3*, to filter the protein pairs from the bottom-N predictions which induce no L3 path in the PPI network. Hence, we obtain the Topological PPNI (TPPNI), which is used in the training and testing of UPNA-PPI. (B) Hyperbolic embeddings have recently been used for visualizing and link prediction tasks in complementarity-driven networks like PPI networks. We visualize the proteins involved in PPI and different PPNIs in the hyperbolic space. We observe that the proteins involved in subcellular compartmental negatives (SCN) and PPNI from the Negatome database show a large overlap with proteins in the PPI network. However, the proteins involved in TPPNI show a clear separability from the proteins in the PPI network. Proteins involved in TPPNI are toward the circumference of the hyperbolic disc and are hence constituted by evolutionarily younger proteins. (C) Lobato *et al*. (2018) established the relationship between the radial coordinates ***r*** of human proteins and their age by assigning proteins to six different age groups through grouping proteins based on their ancient relatives in other species (subfigure courtesy of Lobato *et al*. 2018). The clear selection of younger proteins by *CL3* hypothesis indicates that *CL3* can only identify negative interactions for younger proteins, which are less central in biological pathways. Hence, biology would optimize less competition between the complementarity mechanisms to form a biologically relevant function for younger proteins, leaving patterns in PPI topology that can distinguish younger and older proteins as captured by *CL3*.

Negative sampling is an inextricable part of a binary classification task in machine learning (Chen *et al.* 2020a). The lack of high-quality hard negatives in biological data is a major hindrance in developing practical ML models. The majority of the biological databases have a sufficient number of positive examples, but a considerably low number of negative samples due to experimental and observational biases (Chatterjee *et al.* 2023a), which is a major cause of both overall class imbalance and node-wise class imbalance (annotation imbalance) (Chatterjee *et al.* 2023c). Random negative sampling is the most frequently used approach to mitigate this problem. Yet, random negative samples are consequential in closed systems (Gallaire and Minker 1978) and are prone to degree bias (Chatterjee *et al.* 2023c, Ju *et al.* 2023). In this section, we propose a novel method of creating hard PPNIs by leveraging the PPI network topology (Kovács *et al.* 2019), which is underpinned by the complementary nature of the interactome (Budel and Kitsak 2023). We propose Topological Protein–Protein NonInteraction (TPPNI, see Fig. 2A) that combines entropy-based network null models (Barabási 2016) and higher-order PPI network properties (Kovács *et al.* 2019) in prioritizing hard PPNI samples. TPPNI methodology consists of two parts: (a) a traditional unipartite configuration model, and (b) contrastive-L3 (CL3) filtering.

We use the traditional unipartite configuration model (see Methods), which takes as input only the degree sequence of the PPI network and achieves a commendable performance in transductive link prediction [AUROC: 0.87±0.0002, AUPRC: 0.87±0.0002, Hits@Top100 (Precision at top *K* predictions with *K* = 100): 0.98±0.016]. The output of the configuration model dictates the interaction probability between two proteins from a network topological standpoint. We select the bottom-*N* (*N* = 10 million) predictions from this configuration model on all human protein pairs and consider them as less probable for binding, a.k.a, potential hard negative samples. Next, we use a complementarity-driven topological property of the PPI network to derive the hard negatives. Unlike the formation of triadic closure in social networks (Bianconi *et al.* 2014), proteins tend to form quadratic closures or even-length cycles (Kovács *et al.* 2019). Two friends in a social network have multiple common friends, and hence many paths of length 2 (L2). On the other hand, two interacting proteins have many paths of length 3 or L3 between them, which is related to the evolutionary aspects and the complementary nature of PPIs (Kovács *et al.* 2019, Budel and Kitsak 2023). The L3 hypothesis states the existence of odd-length (*L* = 3) paths in the PPI, i.e. interacting protein–protein pairs induce many L3 paths in the PPI network. We propose the *Contrastive-L3* (CL3) hypothesis (see Fig. 2A), which states that two noninteracting proteins would not constitute any odd-length (*L* = 3) path in the PPI. We now use the CL3 hypothesis to filter high-quality hard negatives from the output of the configuration model. We filter the output of the configuration model mentioned above to fetch the protein pairs that induce no L3 path in the PPI and obtain 3 063 605 hard negatives (see Fig. 2A). Note that, ∼18 000 human proteins in different PPI databases can computationally create ∼156 million potential pairs. This is a computationally infeasible space for computing all L3 paths. Therefore, adding the noninteraction prediction from the configuration model significantly reduces the L3 computation space and provides a regime consisting of proteins lacking sufficient annotations, constituting the potential space for sampling hard PPNIs.

### 3.2 Geometric and evolutionary relevance of PPNI

Recent studies have demonstrated the ability of hyperbolic embeddings to capture evolutionary patterns driven by complementarity in PPIs (Lobato *et al.* 2018). Figure 2 illustrates various PPNI sampling methods in a 2D hyperbolic space (Lobato *et al.* 2018, Kitsak *et al.* 2020). We use the NetHypGeom hyperbolic embedder proposed by Lobato *et al.* (2018). In a 2D hyperbolic plane, the “radial coordinate” represents the hyperbolic distance of a point from a fixed center point, similar to the radius in a Euclidean polar coordinate system, while the “angular coordinate” denotes the angle between a reference direction and the line connecting the center point to the given point, essentially acting like the angle in a polar coordinate system; however, due to the curvature of the hyperbolic plane, the radial coordinate behaves differently than in Euclidean geometry, with distances exponentially increasing as you move further from the center. In Fig. 2B, we visualize proteins involved in Subcellular Compartment Negatives (Jansen *et al.* 2003, Jansen and Gerstein 2004) (SCN, see Supplementary Fig. S1), Negatome 2.0 (Blohm *et al.* 2013), and TPPNIs involving distinct proteins. Lobato *et al.* (2018) have associated the radial and angular coordinates of the 2D hyperbolic plane with the evolutionary aspects of human proteins. According to their findings, proteins closer to the origin are evolutionarily older, while those with higher radial coordinates are younger (see Fig. 2C). We observe that SCN PPNI involves both old and new proteins due to constraints related to subcellular localization. Negatome PPNI is primarily constrained by well-studied older proteins. In contrast, TPPNI is constrained to younger proteins. From an evolutionary standpoint, biological pathways have evolved around older proteins, resulting in their interactions with the majority of other proteins (Zhang *et al.* 2015, Lobato *et al.* 2018). Conversely, younger proteins exhibit fewer interactions among themselves. Moreover, protein families are linked to angular coordinates within the hyperbolic space, as indicated by research on latent geometry in PPNI networks (Lobato *et al.* 2018). The TPPNI encompasses the entire range of angular coordinates, thereby encompassing samples from diverse human protein families, as illustrated in Fig. 2B.

**Figure 3.**
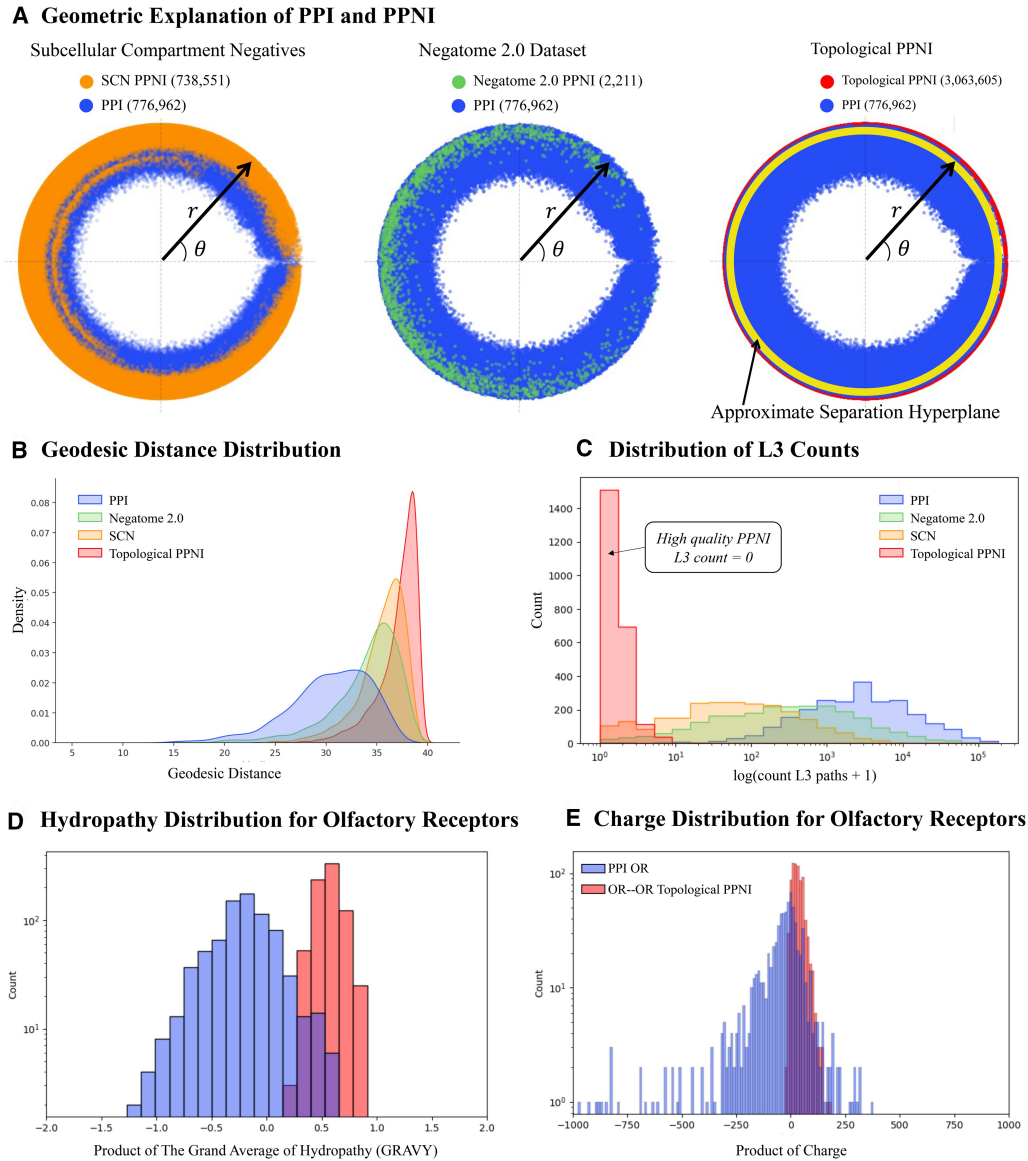
Geometric and chemical relevance of topological PPNI (TPPNI). (A) We visualize each PPI and PPNI in the hyperbolic space by averaging ***r*** and θ for each protein pair. TPPNI provides the best-separating hyperplane that can be learned by downstream tasks only from the secondary structure of proteins. (B) We plot the distributions of geodesic distances between the protein pairs in PPI and different PPNIs (number of samples for each type *n *=* *2311). TPPNI identifies pairs of proteins that are significantly far from each other in the hyperbolic geometry of the human PPI network. Hence, compared to SCN and Negatome, TPPNI offers negative pairs with unique patterns, allowing ML to decode and learn such patterns. (C) We plot the L3 count distributions for PPI and various PPNIs. While the protein pairs in PPNI induce a significantly lower number of L3 paths in the PPI network, we select the pairs inducing no L3 path in the TPPNI for training and testing of UPNA-PPI. (D) The Grand Average of Hydropathy (GRAVY) is a measure of the hydrophobicity or hydrophilicity of a peptide or protein (Kyte and Doolittle 1982). The more negative the score, the more hydrophilic the amino acid is, and the more positive the score, the more hydrophobic. Humans have approximately 400 olfactory receptors (ORs), and we observed 7076 negative inter-family links among ORs in TPPNI. Also, we have 773 positive links between ORs and other proteins in our PPI; none of the 773 PPI OR links are inter-family. The distribution of the product of GRAVY scores between PPI ORs and OR–OR PPNI (randomly sampled 773 TPPNI) shows that the *CL3* resulted in identifying pairs of ORs that show higher hydrophobic mismatch, and hence decrease the chance of interacting with each other. (E) Similarly, we calculated the product of charge for the 773 PPI OR links and OR–OR TPPNI (charge at pH=7). The distribution of the product of charge shows that *CL3* identified inter-family OR proteins with the same sign of charge, hence repelling each other and decreasing the chance of interaction.

In Fig. 3A, we present a visualization of PPI and PPNI edges in a 2D hyperbolic space. The computation of the edge (both PPI and PPNI) embedding between two proteins involves averaging the hyperbolic embeddings of the respective end proteins across each dimension. The substantial overlap observed between SCN PPNI and the experimental PPI implies that SCN fails to effectively capture the complementarity mechanism influencing protein interactions (Budel and Kitsak 2023). Consequently, SCN PPNI proves inadequate for training a classifier capable of distinguishing between protein interactions and noninteractions based on complementarity. A parallel observation is made for Negatome PPNI. Conversely, TPPNI demonstrates effective separation between PPI and PPNI in the hyperbolic space, successfully capturing complementarity-driven interaction mechanisms through negative samples. The hyperplane learned by UPNA-PPI, visualized in Fig. 3A, effectively distinguishes PPI from PPNI in the hyperbolic space. While training a classifier on the hyperbolic embeddings would limit the complementarity-driven link prediction to the transductive scenario and would not be able to make PPI predictions for new proteins, UPNA-PPI demonstrates the ability to learn complementary mechanisms of protein interactions from the amino acid sequences when trained using experimental PPI and TPPNI, and can generalize the learning to new, unseen proteins in inductive tests, hence improving both generalizability and biological interpretability of PPI prediction.

In Fig. 3B, we visualize the distributions of pairwise geodesic distances (Budel and Kitsak 2023) between protein pairs for PPI and various PPNIs. Notably, the pairwise distance distribution for TPPNI is distinctly separated and positioned to the right of the distribution for PPIs. This indicates that, in the hyperbolic space, interacting proteins are geodesically closer compared to those in TPPNI. Moreover, TPPNI demonstrates superior separability between noninteracting proteins compared to SCN and Negatome.

Figure 3C illustrates the L3 paths generated by pairs in PPI and various PPNI scenarios. The visual representation reveals that both SCN and Negatome PPNI induce a significantly lower number of L3 paths in the PPI network when compared to experimentally validated PPIs. To identify TPPNI, we focus on protein pairs that do not induce any L3 paths in the PPI network. Significantly, given that both L3 paths and the hyperbolic space capture complementarity-driven mechanisms in PPI, protein pairs in the TPPNI category, which induce no L3 paths, are positioned farthest away in the hyperbolic space. This distinct positioning underscores their clear separation from PPI pairs that exhibit the highest number of induced L3 paths, providing a distinctive characterization.

Finally, we explore a case study involving human proteins to illustrate how topological negatives effectively capture complementary interaction mechanisms. Canonical Olfactory Receptors (ORs) represent a class of G-protein-coupled receptors (GPCRs) in mammals (Buck 2000). Monomeric ORs in mammals are activated by chemical ligands and couple to specific G-proteins. For example, ORs in the olfactory sensory epithelium activate Cyclic adenosine monophosphate (cAMP) and other second messenger signaling, leading to ion channel opening and membrane depolarization. Contrastingly, insect ORs function as heteromeric ion channels and lack homology to G-protein-coupled chemosensory receptors found in vertebrates (Sato *et al.* 2008, Kaupp 2010). Humans possess >400 ORs, while rodents have approximately 1000 OR genes (Lee *et al.* 2019). Hence, it is biologically justified to observe a higher number of noninteractions between human OR proteins to other human proteins, which is reflected in our TPPNI samples. We observe a total of ∼15 000 topological negatives involving the human OR proteins, among which ∼7000 samples include proteins from inter-OR families. Furthermore, biological justification suggests that TPPNIs are dependent on the species under consideration. Finally, we validate the association between OR-relevant TPPNI and complementarity mechanisms. In Fig. 3D and E, we visualize the distribution of the product of hydropathy (Kyte and Doolittle 1982, Cock *et al.* 2009) and electrostatic charge (Cock *et al.* 2009) for human OR protein pairs in both PPI and TPPNI. Divergent distributions, with the PPNI distributions being more positive, validate that TPPNI effectively captures hydrophobic mismatch and electrostatic complementarity mechanisms (Budel and Kitsak 2023).

### 3.3 UPNA-PPI

Now we use our topological negatives (PPNIs) to design UPNA-PPI, a generalizable, robust, interpretable, and transferable PPI prediction ML pipeline. UPNA-PPI uses unsupervised pre-training of node attributes for improved generalizability to unseen proteins (Chatterjee *et al.* 2023b).

#### 3.3.1 UPNA-PPI architecture

**Figure 4.**
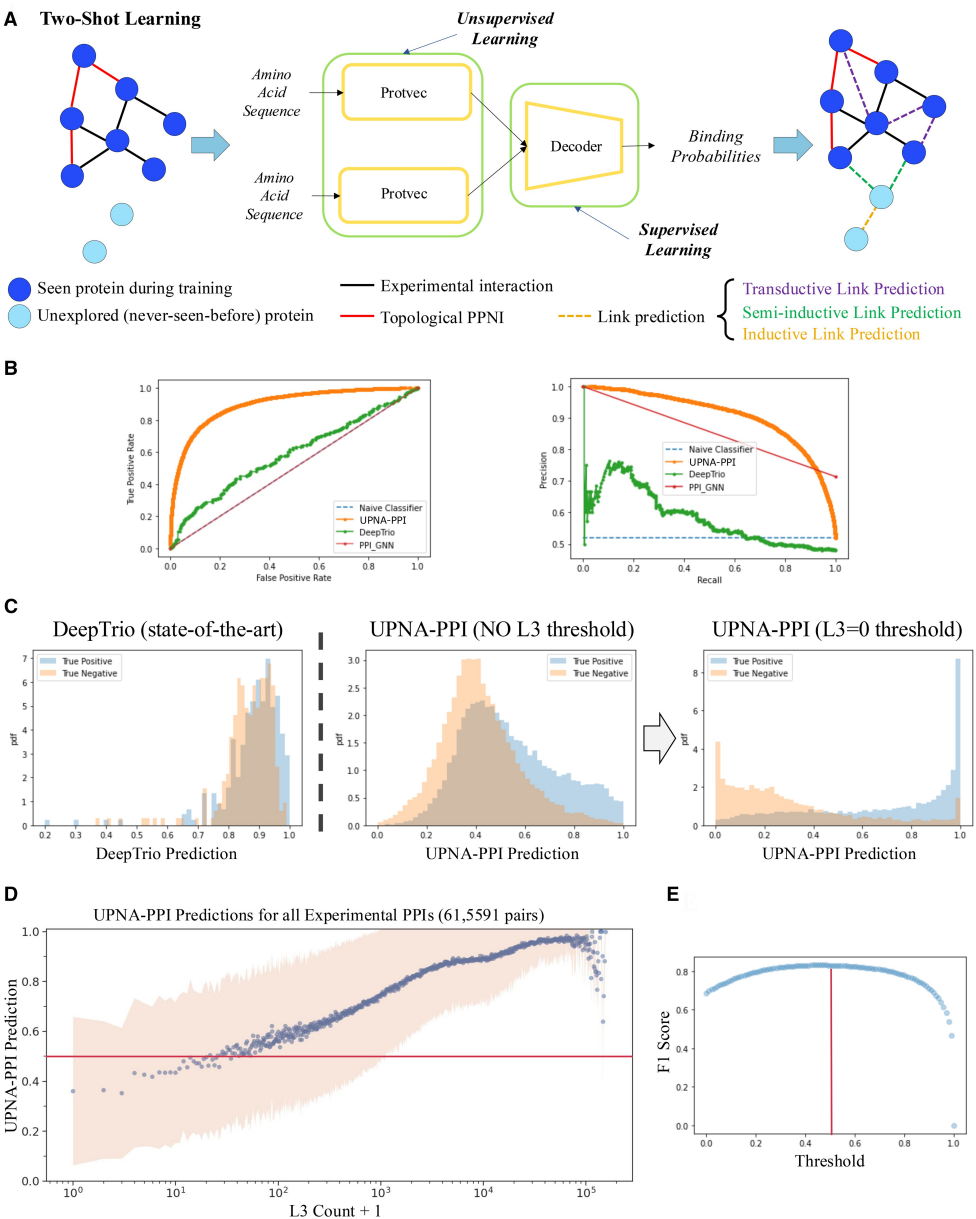
UPNA-PPI architecture, inductive link prediction, and transfer learning. (A) UPNA-PPI architecture: UPNA-PPI embeds the protein amino acid sequences into 100D vectors using ProtVec. For each protein pair, UPNA-PPI concatenates the ProtVec embeddings and feeds them to a decoder (3-layered multi-layer perceptron). (B) UPNA-PPI achieves better performance both in terms of receiver operating characteristics and precision–recall compared to two state-of-the-art models, PPI-GNN and DeepTrio, in inductive link prediction. (C) DeepTrio predicts overlapping prediction values for the PPI and PPNI test samples. UPNA-PPI shows better separation between the predictions for PPIs and PPNIs, becoming a superior ranking tool for both PPI and PPNI. (C) Predictions at the output of DeepTrio are overlapping for the PPI and PPNI data. Hence, DeepTrio is unable to separate the predictions from PPIs versus PPNIs. UPNA-PPI trained on potential negatives from the configuration model (no *CL3*) shows better separation between the predictions for PPIs versus PPNIs. Finally, after introducing CL3 thresholding, UPNA-PPI shows a clear separation between the predictions for PPIs and PPNIs. (D) We computed the number of L3 simple paths between all PPI pairs to investigate if UPNA-PPI has learned the complementarity mechanisms from protein sequences. Indeed, we observe a strong Spearman’s rank correlation coefficient of 0.48 between L3 counts and UPNA-PPI predictions, indicating that the TPPNI enforced UPNA-PPI to learn complementary mechanisms that drive protein–protein interactions only from the amino acid sequences. (E) We plot the F1-score on the test dataset (first fold) by changing the binary classification threshold. We observe that the test F1-score is maximized for an optimal classification threshold ≈0.5. Furthermore, in subplot (D), we observe that UPNA-PPI predicts interaction probabilities greater than the optimal interaction threshold for the majority of the experimentally validated PPIs. Similar observations are made for other folds of UPNA-PPI, and the average optimal threshold is 0.476±0.049.

UPNA-PPI uses pair-wise learning (Ying and Zhou 2015) combined with protein representations pre-trained in an unsupervised fashion (Chatterjee *et al.* 2023b) for improving the generalizability of ML-based PPI prediction. Figure 4A visualizes the neural architecture of UPNA-PPI. We use ProtVec (Asgari and Mofrad 2015) for embedding the protein amino acid sequences. ProtVec is trained on ∼0.5 million amino acid sequences (1.6 million trigram sequences) available in SwissProt (Bairoch 1996).

Although machine learning (ML)-based PPI prediction has been investigated using 3D protein structures (Jha and Saha 2020, Liu and Li 2024), recent insights into the dynamic and self-organizing nature of the cell criticize approaches that consider the rigid structures of proteins (Nicholson 2019). The limitations of the current ML PPI prediction models, stemming from the scarcity of experimental 3D human protein structures and the uncertainties associated with AlphaFold (Callaway 2022), impede achieving high accuracy, robustness, and generalizability. The assumed rigidity of 3D structures fundamentally constrains the predictive power of PPI prediction models. Therefore, we opt to utilize amino acid sequences as a basis for learning PPI from protein representations, aiming to overcome these limitations and enhance the predictive capabilities (Nicholson 2019, Jha and Saha 2020, Callaway 2022, Liu and Li 2024).

The ProtVec embeddings are fed into two arms of the downstream UPNA-PPI decoder, which formulates a binary classification task. We use a 3-layer multi-layer perceptron (MLP) (Haykin 1994) as the decoder. UPNA-PPI is trained in an inductive setting (see Supplementary Fig. S2) (Chatterjee *et al.* 2023b). We use the GoldPPI (see Methods) and Negatome PPNI data in the validation and test datasets for gaining uncompromising confidence in UPNA-PPI predictions through harder tests. Following the inductive link prediction setup from GraIL (Teru *et al.* 2020), the human proteins are randomly divided into three groups for train, validation, and testing, with the proteins from the GoldPPI and Negatome PPNI predominantly residing in the validation and test groups. Then, the interactions induced by these proteins are sampled to create the train, validation, and test datasets. We implemented an early-stopping (Prechelt 2012) on the inductive validation dataset to avoid overfitting. UPNA-PPI is trained and tested in a 5-fold cross-validation setting.

#### 3.3.2 Inductive link prediction and transfer learning performances

Two state-of-the-art PPI prediction models, DeepTrio (Hu *et al.* 2021b) and PPI-GNN (Jha *et al.* 2022) perform exceptionally well in transductive link prediction (see Supplementary Section S2). Yet, their performance significantly diminishes in inductive tests (see Supplementary Table S1, Supplementary Fig. S3). In inductive link prediction, PPI-GNN and DeepTrio achieve significantly lower performance than UPNA-PPI (see Table 1, Fig. 4B). Furthermore, we borrow two performance metrics from recommender systems into PPI prediction to assess the quality of the ranking made by PPI prediction models. We define PPIHits@TopK as the number of true interactions (precision) identified by an ML model in the top K predictions. We also define PPNIHits@BottomK, which quantifies the number of true noninteractions identified in the bottom K predictions of a model. These two metrics bolster our confidence in the ranking provided by the model, which is associated with the separability of the predictions for the PPI and PPNI in test data. UPNA-PPI establishes itself as an excellent ranking tool for identifying PPI in terms of both metrics (PPIHits@Top1000: 0.92±0.01 and PPNIHits@Bottom1000: 0.96±0.02). In Fig. 4C, we observe how DeepTrio creates overlapping predictions for the true interactions and true noninteractions. The incorporation of PPNI at the output of the configuration model, combined with pair-wise learning, improves the separation between these distributions and helps UPNA-PPI predict the true interactions with higher probabilities. Finally, filtering the PPNI with L3 counts (i.e. TPPNI) separates the UPNA-PPI output distributions, confirming the ability of UPNA-PPI to learn the latent PPI patterns driven by various molecular complementarity mechanisms. These observations provide an ablation study for the TPPNI and showcase the strength of the CL3 approach in creating hard negatives (Fig. 4D and E).

**Table 1.** State-of-the-art PPI prediction models versus UPNA-PPI in inductive link prediction.^a^

Model	AUROC	AUPRC	PPIHits@Top100	PPIHits@Top1k	PPIHits@Top10k
UPNA-PPI	0.79±0.01	0.87±0.01	0.98±0.02	0.92±0.01	0.87±0.01
Random Neg	0.74±0.03	0.67±0.04	0.70±0.36	0.86±0.06	0.71±0.05
PPI-GNN	0.50±0.0	0.74±0.03	0.98±0.01	0.67±0.07	0.06±0.006
DeepTrio	0.59±0.06	0.62±0.07	0.67±0.09	0.25±0.008	0.024±0.001

Model	PPNIHits@Bottom100	PPNIHits@Bottom1k	PPNIHits@Bottom10k
UPNA-PPI	0.95±0.03	0.96±0.02	0.88±0.01
Random Neg	1.0±0.0	0.97±0.02	0.82±0.04
PPI-GNN	0.13±0.13	0.23±0.03	0.02±0.004
DeepTrio	0.58±0.07	0.23±0.02	0.2±0.004

a Our proposed pipeline UPNA-PPI, combining TPPNI and unsupervised pre-training on protein representations, significantly improves inductive link prediction performance in PPIs compared to two state-of-the-art PPI prediction models PPI-GNN, DeepTrio, and UPNA’s counterpart with random negative sampling (see Methods for the dataset used to produce this table).

On a similar token, the recent discovery of shortcut learning (Geirhos *et al.* 2020) has raised many questions about the reliability of ML predictions, and inductive tests have gained significant attention in generalizability and interoperability (Chatterjee *et al.* 2023b,c). RAPPPID (Szymborski and Emad 2022) is a recently proposed PPI prediction model that uses inductive learning to improve the generalizability of PPI prediction. RAPPPID uses sequential neural models (LSTM) (Hochreiter and Schmidhuber 1997) to embed the protein amino acid sequences and feed them to a downstream decoder, which is trained for an inductive setting in an end-to-end fashion. Szymborski and Emad (2022) built a novel protein-ligand dataset using BioLiP (Yang *et al.* 2012) (curated PDB interactions). Their dataset utilizes high-quality sequences from X-ray crystallography (Zenodo: https://doi.org/10.5281/zenodo.6709790), offering distinct modalities and interaction biases compared to STRING/UniprotKB (Consortium 2020). Furthermore, BioLiP captures interactions distinct from STRING. While STRING focuses on broad protein classes, BioLiP, derived from X-ray crystallography, biases toward slower, nonaliphatic interactions (Carpenter *et al.* 2008). This distinction makes BioLiP ideal for showcasing transfer learning with RAPPPID and UPNA-PPI. However, the regularization of the protein embeddings in RAPPPID limits the generalizability of RAPPPID within certain protein datasets and protein classes. Performance of RAPPPID significantly reduces in a transfer learning scenario from STRING (Szklarczyk *et al.* 2021) to BioLip (Yang *et al.* 2012). In Table 2, we see that UPNA-PPI achieves comparable performance to RAPPPID for inductive link prediction within similar protein families, while performing better than RAPPPID in a transfer learning setting from STRING to BioLip. Data-specific regularization hinders RAPPID from achieving superior generalizability across datasets and protein families, which is resolved in UPNA-PPI by unregularized and unsupervised protein representations, independent of the training dataset.

**Table 2.** UPNA-PPI versus RAPPPID in inductive link prediction and transfer learning across protein families and peptides.^a^

Model	Inductive				Transfer learning
	AUROC	AUPRC	Hits@Top1000	Hits@Top10 000	AUROC
UPNA-PPI	0.89	0.90	0.98	0.93	0.73
RAPPPID	0.80	0.81	0.77	0.13	0.55

a RAPPPID is an inductive PPI prediction model that uses sequential models and regularization for protein representations. Although UPNA-PPI and RAPPID achieve comparable performances in inductive link prediction, UPNA-PPI offers stronger prioritization as depicted by Hits@TopK. Furthermore, in the transfer learning setting of training on STRING DB and testing on BioLip, which contains proteins from different families than STRING DB, RAPPPID fails and achieves performance similar to a naive Bayes classifier on unseen data (we used the dataset released by RAPPPID to produce this table). However, UPNA-PPI can make meaningful PPI predictions in this transfer learning setting, showing improved generalizability across protein families.

In Fig. 4D, we plot the UPNA-PPI prediction for all experimentally validated PPIs against the number of L3 paths induced by these pairs in the PPI network. A robust correlation is evident between the UPNA-PPI predictions and the L3 counts, with $r_{\mathrm{Spearman}}=0.48$. This observation confirms that UPNA-PPI effectively captures the complementarity-driven mechanism underlying L3 in PPIs based on protein amino acid sequences. Moreover, as depicted in Fig. 4E, the UPNA-PPI predictions for the majority of the experimentally validated PPIs surpass the optimal classification threshold (0.476±0.049).

### 3.4 Comparison with random negative sampling

**Figure 5.**
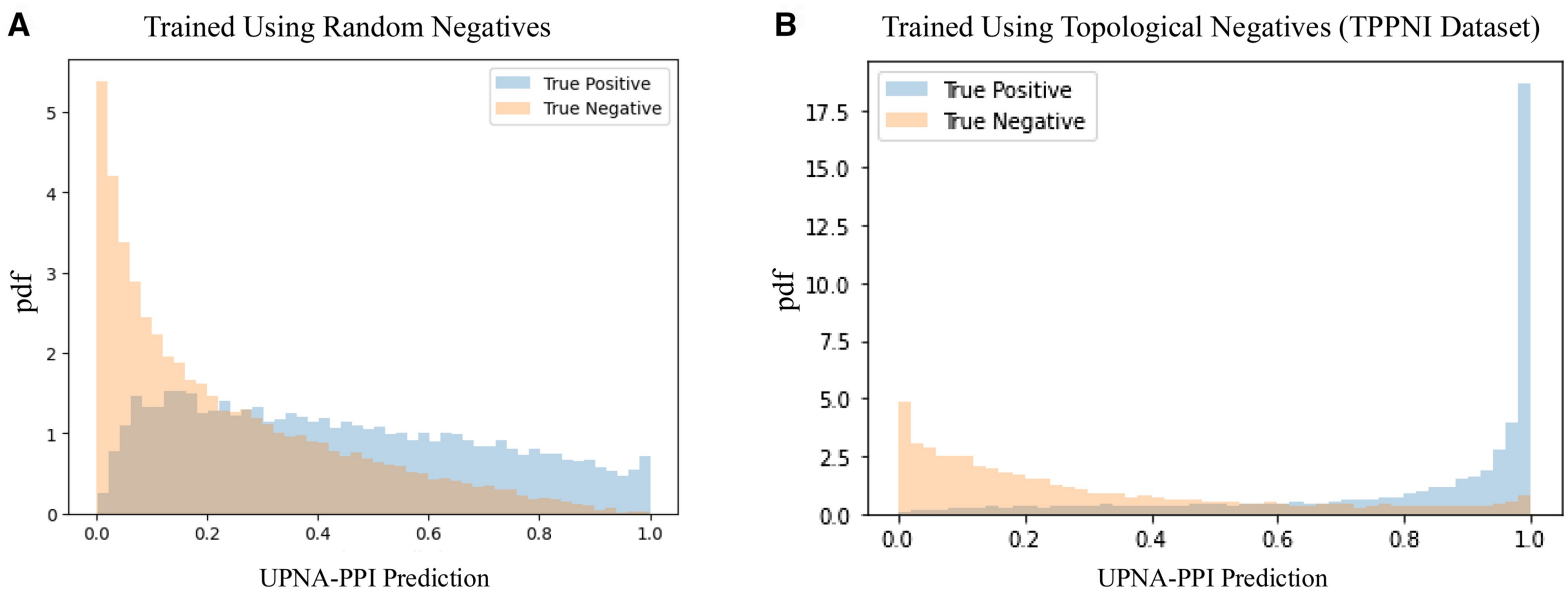
Comparison of UPNA-PPI predictions using random negative samples versus topological negatives. Distributions of predictions for true positives and true negatives in 1-fold of the 5-fold cross-validation datasets. Random negative samples are generated by sampling edges from the complementary graph of the PPI network, maintaining the same number of random negatives as UPNA-PPI samples in the train and validation datasets, while the test dataset remains unchanged (containing complementarity-driven hard negatives and experimental negatives from Negatome 2.0 and NVDT). The figure shows reduced separability (greater overlap) between true positives and true negatives when (A) random negatives are used compared to (B) topological negatives (TPPNI) for training and validation, indicating that the model becomes less confident in distinguishing PPIs from PPNIs.

To investigate the impact of our TPPNI dataset on the performance of UPNA-PPI model, we create random negative samples to use in train and validation datasets by randomly sampling edges from the complementary graph of the PPI network and keeping the number of random negative samples the same as that of the TPPNI samples. We kept the same test datasets as we used for evaluating the UPNA-PPI model, ensuring the same subset of our PPNI dataset is used as it contains both complementarity-driven hard negatives (i.e. TPPNI) and experimental negatives from Negatome 2.0 and NVDT (see Methods). In Fig. 5 (for 1-fold of the 5-fold cross-validation datasets), we observe that the distributions of predictions for the true positives and true negatives are less separable (i.e. more overlapped) when we use random negatives in train and validation sets, i.e. the trained model is less confident of separating the PPI from PPNI. Furthermore, we compare the performances of random negative samples with UPNA-PPNI on inductive tests in Table 1.

### 3.5 Robustness and sensitivity of UPNA-PPI

The robustness of link prediction with the introduction of noise and adversarial attacks in the network provides insight into the reliability of machine learning models (Zhang *et al.* 2016, Pezeshkpour *et al.* 2019). Robustness of PPIs have been studied from various aspects, including randomized null models (Maslov and Sneppen 2002), temperature dependence of stable protein complexes (Deeds *et al.* 2007), and the removal of hub proteins (Azevedo and Moreira-Filho 2015). PPI networks are topologically more robust under four common types of perturbations (i) network nodes are randomly removed (failure), (ii) the most connected node is successively removed (attack), (iii) interaction edges are rewired randomly, and (iv) edges are randomly deleted (Huang *et al.* 2005).

**Figure 6.**
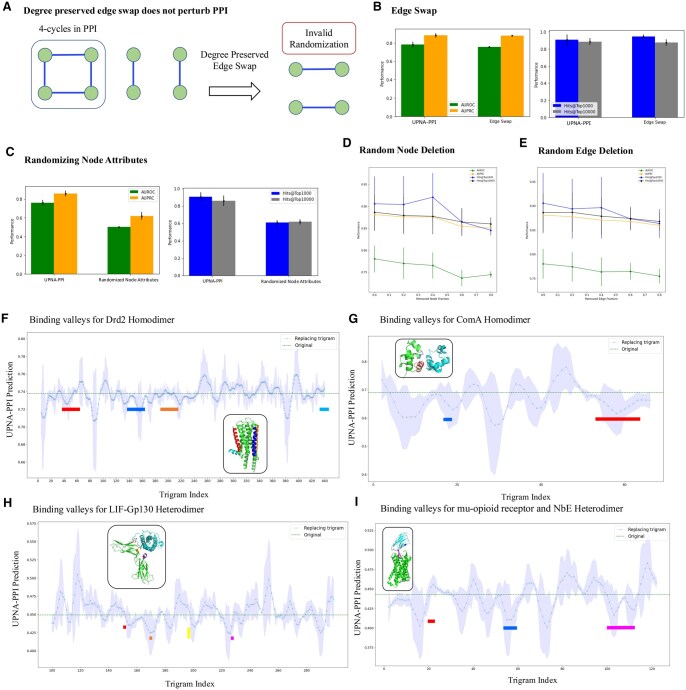
Robustness and Interpretability of UPNA-PPI. (A) Degree preserved edge swap is a widely used method to randomize graphs for studying robustness. However, since PPI networks are enriched with 4-cycles, degree preserved randomization frequently duplicates existing interactions in PPI instead of creating random edges. (B) In the robustness study, we perturb train and validation datasets while keeping the test dataset unchanged. The inductive performance of UPNA-PPI is invariant to degree-preserved randomization. (C) We replace ProtVec embeddings with 100D random vectors where the value for each dimension is randomly selected from a uniform distribution U[0,1]. We observe inductive performance similar to a naive Bayes classifier under such randomization, confirming that UPNA-PPI learns interactions leveraging the embeddings of the amino acid sequences. (D) We randomly remove nodes (proteins) from the training and validation datasets. We do not observe significant fluctuation in UPNA-PPI inductive performance, which confirms the ability of UPNA-PPI to learn from limited data and generalize to new proteins. (E) Similar to (D), we randomly remove edges (interactions) from the training and validation datasets. We do not observe significant fluctuation in UPNA-PPI inductive performance, which confirms the ability of UPNA-PPI to learn from limited data and generalize to new proteins. (F) We ran an ablation study on the amino acid sequence of DRD2 to identify potential trigrams where interaction takes place for creating the Drd2 homodimer. In this ablation study, each amino acid trigram is replaced with the Out-of-Vocabulary (OOV) embedding from ProtVec, while keeping the other amino acid sequence in the input of UPNA-PPI unchanged. We observe multiple valleys in the binding probability profiles. These valleys correspond to the interfeces TM4/TM5 and TM1/H8, which have been identified experimentally using Cys-crosslinking and FRET. (G) We repeat a similar process for another homodimer of transcription factor ComA. (H) and (I) We run an ablation study on two heterodimers consisting of protein pairs LIF-Gp130 and Mu-opioid receptor-NbE. The interaction locations on protein complexes LIF-Gp130 and mu-Opioid receptor-NbEHH are marked in the figures, which overlap with valleys predicted by UPNA-PPI. In all of the above scenarios, we observe that the valleys with lower standard deviation obtained from 5-folds of UPNA-PPI correspond to the true binding locations. Therefore, the valleys on which 5-fold of UPNA-PPI agree are the binding locations with a higher confidence.

In Fig. 6A, we show how L3 paths (4-cycles) in PPI make the networks robust under degree preserved edge swap (Roberts and Coolen 2011) (see Supplementary Section S6 for details). In Fig. 6B, we observe that the inductive link prediction performance of UPNA-PPI does not fluctuate under degree-preserved edge swap in train and validation datasets, while keeping the test data unchanged. This form of robustness is inherent to the PPI network topology, and degree-preserved edge swap is insufficient for evaluating the robustness of any ML-based PPI prediction model.

Next, we test the sensitivity of UPNA-PPI on the protein representations. We replace the 100D ProtVec embeddings with vectors whose entries are drawn at random from a uniform distribution U[0,1]. In Fig. 6C, we observe that UPNA-PPI performance drops significantly when the protein representations are randomized and the performance is comparable to a naive Bayes classifier. This confirms that UPNA-PPI learns PPI mechanisms leveraging the protein amino acid sequences and does not resort to any form of shortcut learning.

Finally, we test the robustness of UPNA-PPI under random node and edge deletion. We perturb the train and validation datasets while keeping the test data unchanged. In Fig. 6D and E, we observe slight decay in the inductive test performance of UPNA-PPI under random deletion of nodes and edges from the training and validation PPI. However, even after deleting 80% of training nodes and edges, UPNA-PPI is still able to achieve commendable inductive link prediction performance. This confirms the high generalizability of the proposed ML pipeline under data scarcity and the power of topological negatives in capturing the complementary mechanisms behind protein interactions.

### 3.6 Interpretability and identifying interaction locations

Finally, the molecular interpretability of PPI predictions is crucial for understanding their biological significance, their role in various cellular processes, and their potential as drug targets. We developed an ML-based approach for identifying putative interaction regions in a given pair of putative interacting proteins by ablating their sequences (Meyes *et al.* 2019). Keeping the amino acid sequence of one protein fixed at a time, we mutate each amino acid trigram of the other protein by replacing it with the Out-of-Vocabulary (OOV) probing with an in-vocabulary example (IVE) entry in ProtVec, and observe the change in UPNA-PPI output prediction probability. Note that, the OOV embedding vector in ProtVec is obtained by the average of all the trigram embedding vectors (Asgari and Mofrad 2015). The valleys of the binding probability profiles correspond to the potential binding sites on the protein sequence. We validate this method on experimentally validated self-interacting protein pairs Drd2 (Guo *et al.* 2008) and ComA (Hobbs *et al.* 2010), and two heterodimeric complexes of proteins: Leukemia inhibitory factor (LIF) in complex with gp130, and mu-opioid receptor bound to NbE. In Fig. 6F–I, we mark the valleys identified by UPNA-PPI which correspond to the binding interfaces identified by experimentally for these complexes. For Drd2 the interacting TM helices have been reported as self-interactions between a region around TM4, and another one in TM1 utilizing Cys-crosslinking and FRET (Guo *et al.* 2008) (Fig. 6F). The transcription factor Competence protein A (ComA) dimerization has been determined by NMR, showing self-interaction mostly on C-terminal helix alpha-10 (Hobbs *et al.* 2010). LIF interaction interface with gp130 was determined by X-ray crystallography (Boulanger *et al.* 2003). Finally, the binding site of NbE on the orthosteric site of the mu-opioid receptor was also determined with X-ray crystallography (Yu *et al.* 2023).

#### 3.6.1 Novel interactions for G protein-coupled receptors

**Figure 7.**
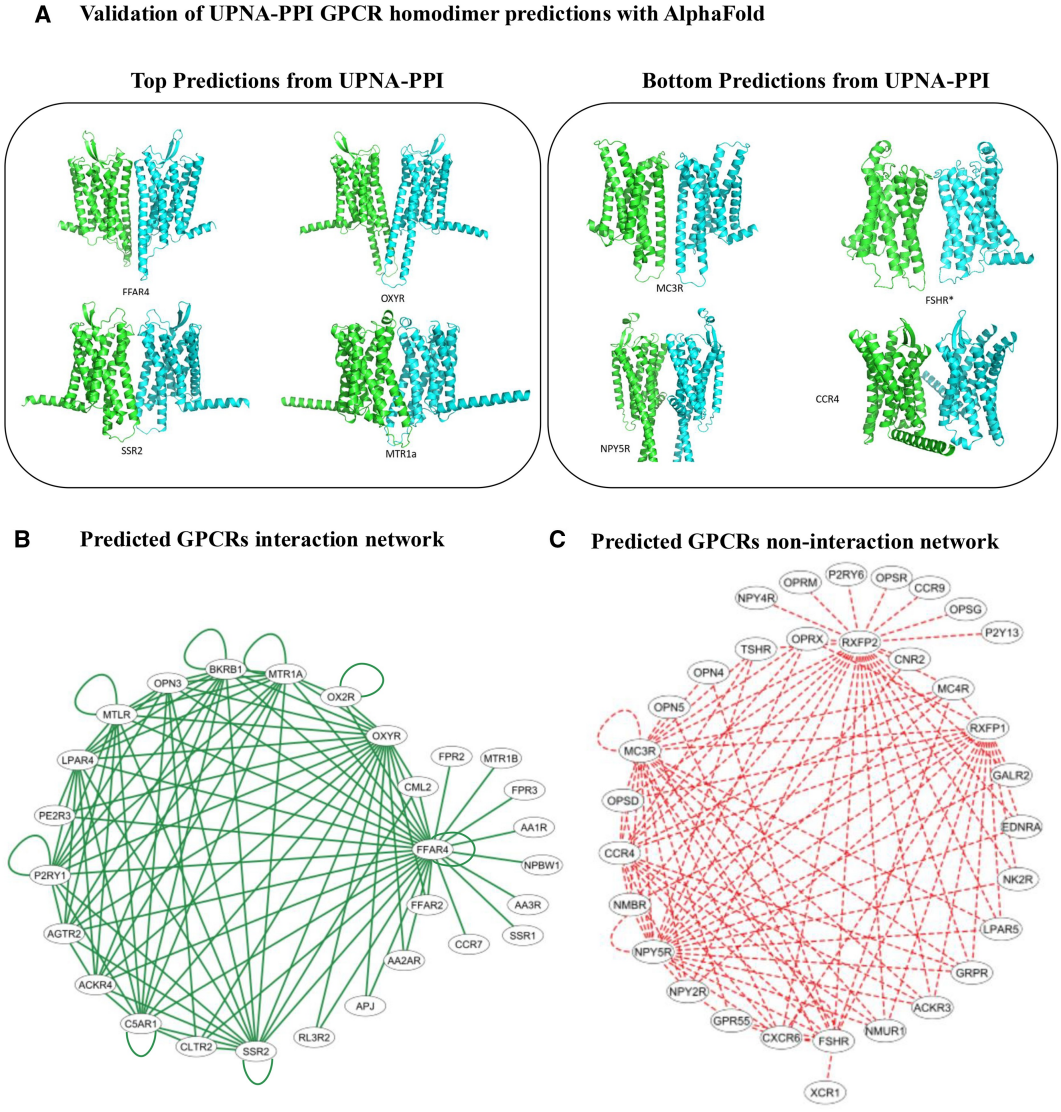
UPNA-PPI Predictions for G-protein coupled receptors (GPCRs). (A) We validate top and bottom self-interaction predictions for GPCRs from UPNA-PPI with AlphaFold-Multimer. We observe that AlphaFold predicts a higher number of atoms at the interaction surface and pLDDT at the surface for the top predictions compared to the bottom predictions (see Table 3), which validates the agreement between UPNA-PPI and AlphaFold. (B) Interaction network of GPCRs. From the top 100 UPNA-PPI predictions, we construct the interaction network of GPCRs. We predict 8 homodimers and 92 heterodimers involving 28 GPCR proteins. (C) Similarly, we construct a noninteraction network with the bottom 100 predictions from UPNA-PPI.

Physiologically and pharmacologically relevant G-protein-coupled receptors (GPCRs) are the target of several marketed drugs (Rosenbaum *et al.* 2009). They mediate physiological responses to hormones, neurotransmitters, and environmental stimuli. Recent high-resolution structural studies have shed light on the molecular mechanisms of GPCR activation and constitutive activity. Over the past three decades, significant progress has been made in understanding GPCRs, from pharmacology to in vivo function. However, mostly G protein-coupled receptor-G protein interactions have been explored extensively (Calebiro *et al.* 2021). Moreover, accurately predicting PPIs between GPCRs is particularly challenging due to their insertion in the lipidic membrane and the potential role of lipids on those interactions is not captured by docking programs. Chemical-crosslinking experiments have shown GPCR-GPCR self-interactions (dimers and oligomers) on the membrane in cells (Guo *et al.* 2008) while the structural details of those natural interactions have succeeded on limited cases for class A GPCRs (Gusach *et al.* 2023). This is why testing UPNA-PPI on GPCRs cases is a challenging task to test the generalizability of our method and offer insights that may not be available with other methodologies. We used UPNA-PPI to predict potential homodimers and heterodimers between GPCRs. We consider 170 family A GPCR proteins for this prediction task. In Fig. 7, we visualize the GPCR interaction network from the top 100 UPNA-PPI predictions. The network consists of 28 GPCR protein nodes with 8 homodimers and 92 heterodimers. Furthermore, since UPNA-PPI is not trained on self-interactions, we evaluated the homodimeric predicted by UPNA-PPI with AlphaFold-Multimer predicted homodimer structures (Evans *et al.* 2021, Jumper *et al.* 2021). In Table 3, we summarize the AlphaFold average of predicted local distance difference test (pLDDT) score, a measure used by AlphaFold to indicate how confident the AI is in its prediction of a protein’s 3D structure at each specific location (amino acid) along the protein chain, atoms at the interface, and interface pLDDT score for top and bottom homodimer predictions made by UPNA-PPI (see Fig. 7). Segregation of the potential GPCR interactions predicted by UPNA-PPI is in agreement with more computationally expensive results from AlphaFold-Multimer. Hence, UPNA-PPI can be used as a potential high-throughput tool for novel PPI prediction.

**Table 3.** UPNA-PPI validation with AlphaFold multimer.^a^

Case	Model	Average pLDDT	Atoms at interface	Interface pLDDT
Ffar	Rank 0	73.98	358	52.54
Oxyr	Rank 0	71.79	22	63.76
Ssr2	Rank 0	67.09	174	55.6
Mtr	Rank 2	82.24	244	58.54
Average for Top		73.775	199.5	57.61
Mcr	Rank 0	70.42	206	61.99
Fsh	Rank 0	75.28	120	70.34
Npy	Rank 0	59.09	0	0
Ccr	Rank 0	76.67	0	0
Average for bottom		70.365	81.5	33.0825

a While UPNA-PPI is trained on heterodimers, we test the transferability of UPNA-PPI by predicting homodimers. For this experiment, we use understudied GPCR proteins. The top GPCR homodimer predictions from UPNA-PPI include the proteins Fafr, Oxyr, Ssr2, and Mtr. The bottom predictions for self-interaction prediction include the proteins Mcr, Fsh, Npy, and Ccr. We run AlphaFold-Multimer simulations for the listed proteins and summarize the observations in the table below. We observe that, on average, the top self-interaction predictions from UPNA-PPI show more atoms at the interaction interface and higher pLDDT (confidence) at the interface compared to the bottom predictions. Furthermore, while the average pLDDT values are comparable between top and bottom predictions, interface pLDDT is higher for the top predictions, suggesting that UPNA-PPI can learn the interfaces of interaction leveraging only the amino acid sequences.

## 4 Discussion and conclusion

While machine learning has widely been used in PPI prediction, generalizing these models to novel proteins, transferring across protein families, and interpretability of predictions from a molecular standpoint have emerged as the major pitfalls of these models. The scarcity of high-quality, biologically relevant hard PPNI sampling has been a major hindrance for machine learning models to learn interaction mechanisms from protein structural patterns. We have proposed a novel approach to leveraging the PPI network topology in sampling PPNI contingent on the complementarity-driven generation mechanisms of PPIs. We interpret TPPNI geometrically by leveraging the hyperbolic space and making a connection between the complementarity of PPI and the evolution of proteins. Our PPNI sampling approach, combined with unsupervised pre-training of protein representation, not only improves the generalizability of PPI prediction but also improves the transferability of machine learning prediction across protein families. UPNA-PPI is also able to identify potential pocket locations on the amino acid sequences, bolstering the molecular interpretability of machine learning prediction. Furthermore, the robustness of UPNA-PPI under random node and edge removal strengthens the notion of generalizability under data scarcity (Bansal *et al.* 2022).

Complementarity-driven networks induce even-length cycles (Budel and Kitsak 2023). We have developed a novel negative sampling strategy leveraging the 4-cycles in PPIs. This approach can be extended to other complementarity-driven networks by exploring the even-length cycles enriched in the networks. Furthermore, there has been much research in identifying and counting 4-cycles in directed graphs (Eisenbrand and Grandoni 2003, Abboud *et al.* 2022) and sparse graphs (Burkhardt and Harris 2023). Integrating these algorithms to the TPPNI methodology can overcome the need for the configuration model and help us derive more hard PPNI samples. We also hypothesize that PPI networks from different species should be treated individually to create TPPNI. Considering the combined PPI of humans and other species would hinder us from capturing meaningful evolutionary patterns and the complementary nature of the network.

In its exploration of drug-target interaction networks, AI-Bind (Chatterjee *et al.* 2023c) used network science to comprehend topological shortcuts and generate negative samples. In contrast, UPNA-PPI extends the application of network science to ML models operating on PPI networks. UPNA-PPI introduces a novel methodology for negative sampling by leveraging higher-order network properties. Beyond supplying valuable hard negatives applicable to a diverse array of ML models, UPNA-PPI pioneers a new research direction that advocates the use of network topology in negative sampling. This innovative approach holds promise for enhancing the generalizability, robustness, and interpretability of graph machine learning methodologies on a wide range of networks.

## Supplementary Material

btaf148_Supplementary_Data

## Data Availability

We used QIAGEN BKB (Biomedical Knowledge Base) PPI data to obtain experimentally validated PPIs (BKB [2023.2]). BKB is available upon request from QIAGEN (BKB [2023.2]). The predictions and UPNA-PPI codes that support the findings of this study are openly available on GitHub at https://github.com/alxndgb/UPNA-PPI.
