# Supplementary material for: Topology-driven negative sampling enhances generalizability in protein–protein interaction prediction
Source: Bioinformatics. 2025 Apr 7;41(5):btaf148. doi: 10.1093/bioinformatics/btaf148 (PMC12080959; doi:10.1093/bioinformatics/btaf148)
Supplement: btaf148_Supplementary_Data [file btaf148_supplementary_data.pdf]

Supplementary Information

Topology-Driven Negative Sampling Enhances Generalizability in Protein-Protein Interaction Prediction

Ayan Chatterjee[1,2,3,†] , Babak Ravandi[2,3,4,†,*], Parham Haddadi[2], Naomi H. Philip[2], Mario Abdelmessih[2], William R. Mowrey[2], Piero Ricchiuto[2], Yupu Liang[2], Wei Ding[2], Juan C. Mobarec[5], Tina Eliassi-Rad[3,6,7,8]

[1]BioClarity AI, Boston, MA, USA
[2]Alexion AstraZeneca Rare Disease, Boston, MA, USA
[3]Network Science Institute, Northeastern University, Boston, MA, USA
[4]Department of Physics, Northeastern University, Boston, MA, USA
[5]Mechanistic and Structural Biology, Discovery Sciences, R&D, AstraZeneca, Cambridge, UK
[6]Khoury College of Computer Sciences, Northeastern University, Boston, MA, USA
[7]Santa Fe Institute, Santa Fe, NM, USA
[8]The Institute for Experiential AI, Northeastern University, Boston, MA, USA

[†]Ayan Chatterjee and Babak Ravandi equally contributed.
[*]Corresponding author e-mail: babak.ravandi@alexion.com or bk.ravandi@gmail.com

Contents

1 Subcellular Compartmental Negatives (SCN) 2

2 Revisiting Topological Shortcuts in PPI Network 3
2.1 State-of-the-art PPI Prediction Models in Transductive Tests . . . 5
2.2 Sealing Information Leakage, Inductive Tests, and Generalizability . . . 7

3 Unipartite Duplex Configuration Model 8
3.1 Overview . . . 8
3.2 Mathematical Formulation . . . 8

4 Annotation Imbalance 9

5 Fundamental Nature of L3 paths 11

6 Degree-Preserved Edge-Swap in PPIs 11

7 Coefficient of Variation for GPCR Predictions. 12

8 Alternate Protein Embeddings 12

9 Sequence Identities Between the Train and Test Sets 14

10 UPNA Capturing PPI Network Properties 15

1 Subcellular Compartmental Negatives (SCN)

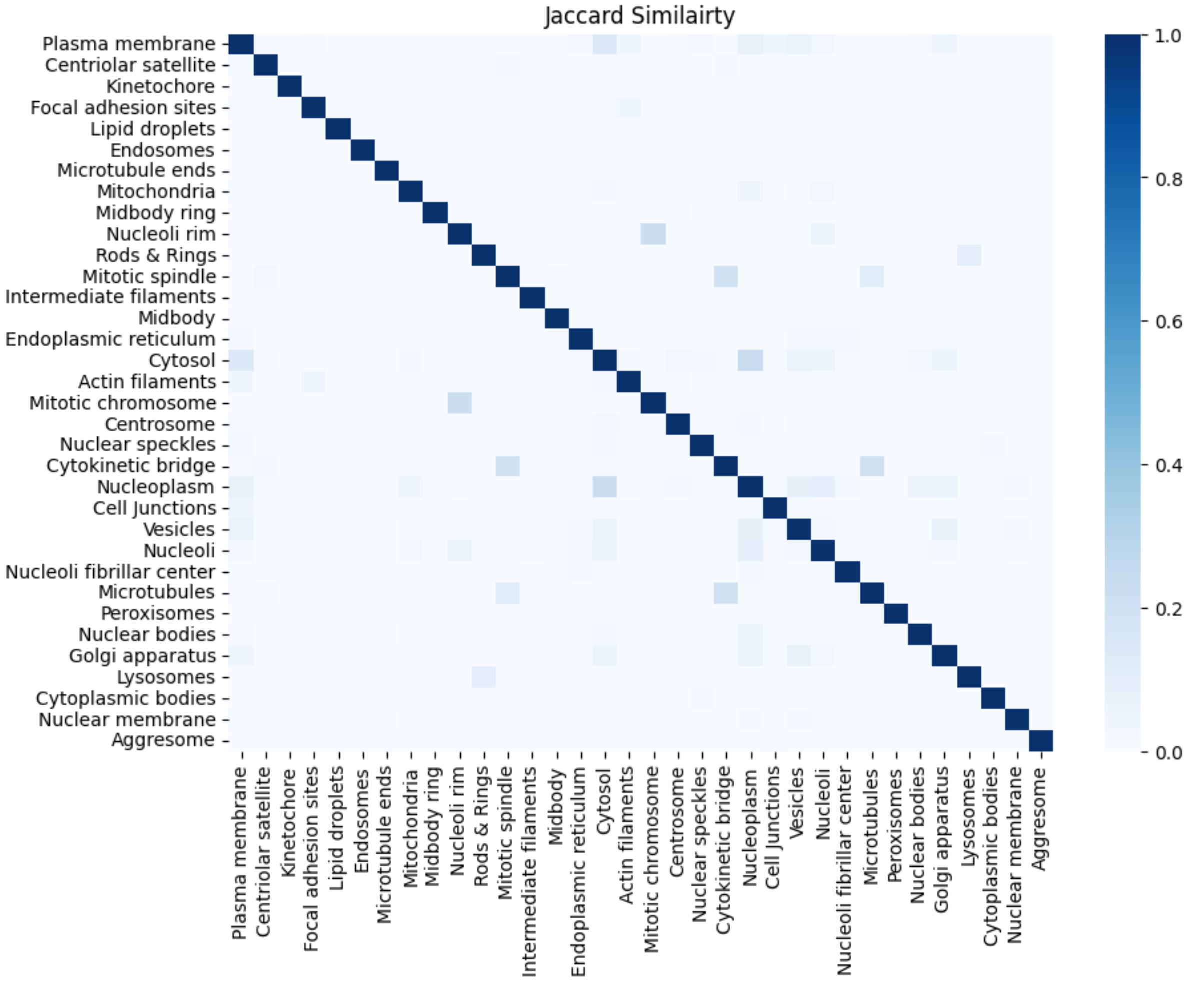

Figure S1: **Subcellular Compartmental Negatives (SCN).** Protein overlap among 34 subcellular compartments in terms of Jaccard similarity [1]. PPNI pairs are samples from a pair of compartments that have no overlapping protein in the heatmap.

2 Revisiting Topological Shortcuts in PPI Network

Most real-world networks, such as Protein-Protein Interaction (PPI) [2] and Drug-Target Interaction (DTI) [3] networks, typically exhibit a degree distribution that follows a heavy-tailed distribution [4]. This means that the majority of nodes in these networks have only a small number of links (interactions or samples) in the training datasets, while a small number of hub nodes have a large number of connections. During the training phase of a link prediction model, the model tends to focus more on these hub nodes than on the nodes with lower degrees. In traditional machine learning scenarios, train and test datasets are created by randomly dividing the edges within the network [5]. As a result, the majority of edges in both the training and test datasets involve these hub nodes. Machine learning models become proficient at learning the neighborhood characteristics of these hub nodes in terms of their degree information [6,7]. Consequently, they can make accurate predictions for the edges associated with these hubs, leading to excellent test performance. Figure S2 demonstrates the need for the inductive scenario in link prediction tasks. The transductive scenario, most frequently used to evaluate the performance of AI models, favors learning topological shortcuts instead of the Mechanisms of Actions (MoA) behind the emergence of structural topology in networked data (Figure S2B). In other words, state-of-the-art (SOTA) models leverage the neighborhood topology of a node (protein or drug) to make new interaction predictions, which are often truant of biological and molecular interpretability. However, in an inductive setting (see Figure S2A), the SOTA models struggle when dealing with the low-degree nodes, since they are forced to leverage the molecular patterns for learning interactions. Hence, hubs play the main role in the misleading high performance of the SOTA models. However, in Erdős-Rényi (ER) graphs [8], there are no hubs, and all nodes are indistinguishable in terms of their neighborhood degrees (see Figure S2B). Thus, link prediction models achieve performance similar to a naive Bayes classifier (AUROC ≈ 0.5) when trained and tested on ER graphs (see SI Section 1 of [6]). Furthermore, AI models trained under transductive scenarios are unable to perform well even in transductive link prediction tasks for low-degree nodes [6]. This highlights the models' limitations in extracting meaningful patterns from the molecular structures of proteins, resulting in poor inductive performance, revealing the misleading nature of transductive tests, and insufficient biological interpretability of the machine learning predictions. Likewise, current machine learning models demonstrate outstanding effectiveness when analyzing common diseases that benefit from abundant data available in established databases [9]. Nonetheless, when it comes to rare diseases [10], the limited data availability restricts the utility of these models in making valuable predictions (see Figure 2C).

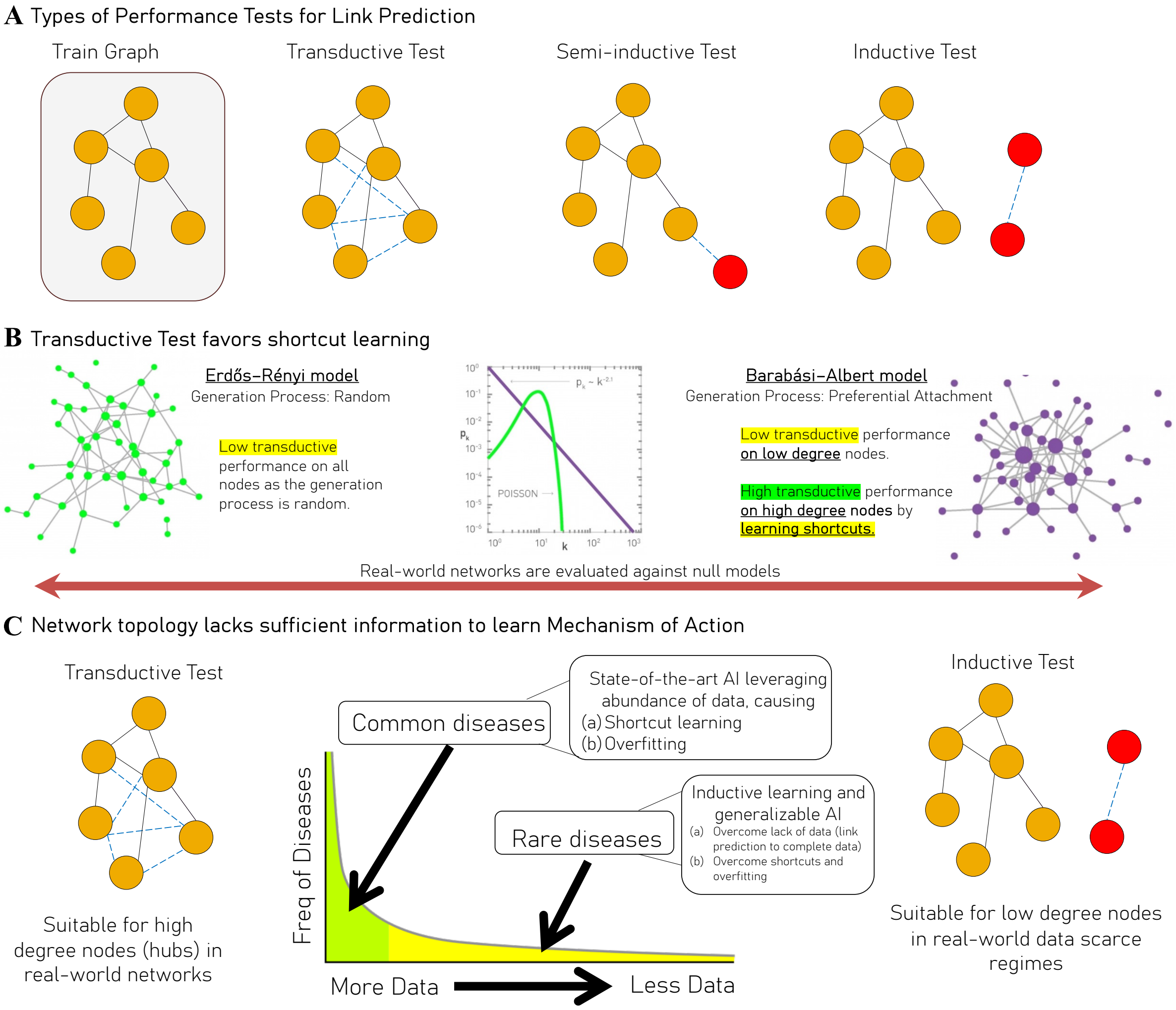
A Types of Performance Tests for Link Prediction
Train Graph
Transductive Test
Semi-inductive Test
Inductive Test
B Transductive Test favors shortcut learning
Erdős–Rényi model
Generation Process: Random
Low transductive performance on all nodes as the generation process is random.
POISSON
Barabási–Albert model
Generation Process: Preferential Attachment
Low transductive performance on low degree nodes.
High transductive performance on high degree nodes by learning shortcuts.
Real-world networks are evaluated against null models
C Network topology lacks sufficient information to learn Mechanism of Action
Transductive Test
Suitable for high degree nodes (hubs) in real-world networks
Common diseases
State-of-the-art AI leveraging abundance of data, causing
(a) Shortcut learning
(b) Overfitting
Rare diseases
Inductive learning and generalizable AI
(a) Overcome lack of data (link prediction to complete data)
(b) Overcome shortcuts and overfitting
Freq of Diseases
More Data
Less Data
Inductive Test
Suitable for low degree nodes in real-world data scarce regimes

Figure S2

Figure S2: **Transductive Test Favors Shortcut Learning.** The selection of test scenario can significantly affect the patterns that ML models learn from data. Intuitively, we expect deep models to learn the Mechanisms of Actions (MoA) behind the generation of data, yet it is common that ML models learn shortcuts instead. **(a)** In transductive scenario both the topological structure (patterns of connections) and node attributes are included in the training phase. The semi-inductive scenario partially hides the topological structure from one side of link prediction, by predicting interaction between a node never-seen-before (annotated with red color) and another node that has been seen during the training phase. Lastly, the inductive test prevents the leakage of topological information from train to test/validation sets, conducting link prediction strictly on data-never-seen before, which enforces an AI to learn MoA from node attributes rather than patterns of connections between nodes (shortcuts). **(b)** Real-world networks are often evaluated against two extremes, one is Erdős–Rényi (ER) model where the graph is generated randomly, and the other is Barabási–Albert (BA) model where the graph is generated by enforcing preferential attachment based on the degree of nodes on each iteration of BA model. In ER, the transductive test performs poorly as the graph is generated purely by randomness. In BA model the transductive test only performs well on high degree needs as the classifier learns high degree nodes have a higher chance of having connections. Yet the classifier does not perform well on low degree nodes since the network topology alone does not have enough information to learn the MoA (preferential attachment), which in this case is the parameters used to generate a BA model. **(c)** Transductive scenario may offer insightful predictions when abundant of data is available by facilitating learning the structural topology. Yet, in the data scarce regime, like rare disease area, the performance of transductive scenario is low due to lack of topological structure. In the data scarce regime, learning from node attributes is essential for link prediction, hence we need to enforce learning the MoA behind emergence of structural topology that is domain specific (e.g., social and biological interactions are driven through different MoAs).

2.1 State-of-the-art PPI Prediction Models in Transductive Tests

Protein-protein interactions (PPIs) exhibit a complex hierarchy of generation mechanisms. At the top level, we have Preferential Attachment (PA) [4], a concept that plays a crucial role in a link prediction model's ability to excel when it comes to predicting connections involving hub proteins. However, it is important to note that PA itself arises as a consequence of certain proteins having a greater number of binding pockets and findings in experimental studies. SOTA models, despite their sophistication, often fall short of understanding the true underlying mechanisms by leveraging the molecular structures of proteins. It's during inductive tests that these models are compelled to uncover this genuine generative process, which, in turn, enhances the model's interpretability. This assertion is substantiated through an experiment involving the introduction of random amino acid sequences onto a DTI network, where the state-of-the-art binding prediction model DeepPurpose [11] model achieves exceptional transductive performance, showcasing the capacity to discern the authentic generation mechanism.

In Figure S3A we visualize the heavy-tailed degree distribution of the QIAGEN BKB PPI [12]. We consider two state-of-the-art PPI prediction models, DeepTrio [13] and PPI-GNN [14]. We develop a unipartite duplex configuration model (see Section S3) [15], which takes as input only the degree sequences from the protein-protein interaction and non-interactions (random negative sampling) used in the training of DeepTrio and PPI-GNN (see Figure S3B). We observe that using only the degree information of the proteins and being completely blind to the protein structures (amino acid sequences), the configuration model achieves comparable performance to the SOTA models (Figure S3C). This experiment confirms that the SOTA models are unable to learn from the protein amino acid sequences and resort to topological shortcuts [6], hence

lack biological interpretability in the predictions.

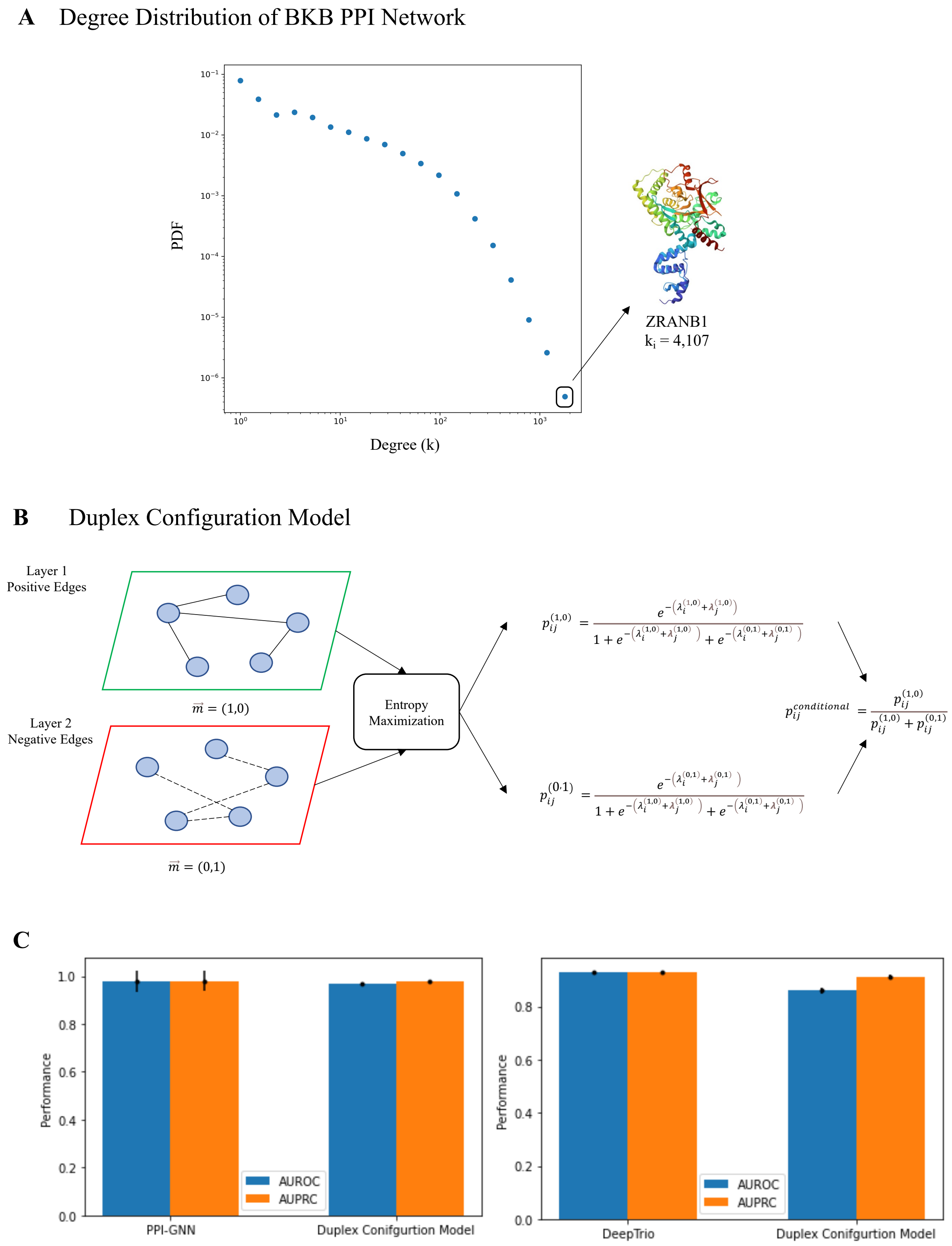

Figure S3

Figure S3: **State-of-the-art and Duplex Configuration Model. (A)** We observe a heavy-tailed degree distribution in the QIAGEN BKB [12] PPI network, which confirms the existence of hubs (high-degree nodes) in the PPI network. For example, ZRANB1 interacts with 4,107 other genes, constituting a hub. **(B)** The unipartite duplex configuration model takes as input PPI training data and random negative samples. By an entropy maximization, we first predict the link probabilities in each layer, which we then combine to obtain the output probability $p_{ij}^{conditional}$. **(C)** The duplex configuration model achieves performance comparable to two state-of-the-art PPI prediction models PPI-GNN and DeepTrio in the transductive setting.

2.2 Sealing Information Leakage, Inductive Tests, and Generalizability

Following the common formulation of GraIL [16], Park et al. [17], and Chatterjee et al. [7,18], we evaluate the performance of the SOTA models in different test scenarios. We have already seen that in the transductive setting, proteins are shared between train and test datasets in a transductive setting (Figure S2A). In a semi-inductive setting, one protein of the test edge is present in train data, and the other one is unseen during training. In an inductive link prediction setting, both proteins of the test edge are absent in training. In Table S1, we observe a major lowering of the test performance of the SOTA models as we move from transductive to inductive tests.

This observation is primarily driven by the reduction of topological information leakage between train and test data. To develop a generalizable AI model, learning PPI from the protein structures instead of leveraging the topology that inflates the performance, we need to mitigate shortcut learning and make protein representation learning independent of the PPI training data. Furthermore, regularization has been proven to be an important tool for improving generalizability in computer vision [19]. Yet, regularization is based on the assumption that similarity between a pair of entities implies interaction, an assumption that is true in image processing and social sciences [20], yet similar molecules do not necessarily always interact in PPI and other biological systems [21]. Although [20] is applicable for identifying similarity between images, the similarity of proteins does not imply their interaction, and hence the regularization-based approach to overcome negative sampling is not applicable in PPI prediction. Thus, we leverage the network topology of the PPI network to create high-quality PPNI for training our AI pipeline.

Table S1: **State-of-the-art PPI Prediction Models in Transductive and Inductive Link Prediction.** In transductive link prediction, nodes are shared between train and test datasets. State-of-the-art PPI prediction models, PPI-GNN and DeepTrio, achieve excellent transductive link prediction performance. However, in inductive tests, when encountered with never-before-seen proteins, the performances of both models diminish significantly.

Model	Transductive		Inductive	
	AUROC	AUPRC	AUROC	AUPRC
PPI-GNN	0.98 ± 0.044	0.98 ± 0.042	0.50 ± 0.00	0.74 ± 0.03
DeepTrio	0.93 ± 0.003	0.94 ± 0.005	0.59 ± 0.06	0.62 ± 0.07

3 Unipartite Duplex Configuration Model

3.1 Overview

Protein-protein annotations naturally form an integral part of a unipartite duplex network, which can be conceptualized as a network comprising a set of nodes representing all proteins. This network operates on two distinct layers, with each layer corresponding to a specific type of interaction occurring between the same pair of nodes, as described in Menichetti et al. [15]. Specifically, Layer 1 characterizes positive interactions, while Layer 2 records negative interactions (see Figure S3B).

The multilink notation, denoted as $\mathbf{m}$, is employed to encode the pattern of links connecting two nodes across different layers. Notably, $\mathbf{m} = (1, 0)$ signifies positive interactions, $\mathbf{m} = (0, 1)$ represents negative interactions, $\mathbf{m} = (0, 0)$ signifies the absence of any interaction, and $\mathbf{m} = (1, 1)$ is mathematically prohibited, as it is impossible for both positive and negative interactions to coexist for the same pair of proteins.

In our analysis, we utilize the canonical unipartite duplex null model, which ensures the conservation, on average, of the number of positive and negative annotations associated with each node. This model also appropriately rewires positive and negative links while avoiding the occurrence of forbidden configurations. By applying entropy maximization with constraints, we derive analytical expressions for the probability of each multilink configuration and the conditional probability of observing a positive binding event once an annotation is reported.

3.2 Mathematical Formulation

Let $A_{ij}^{\mathbf{m}}$ denote the multi-adjacency matrix representing the unipartite duplex network of proteins $\{i\}$ and $\{j\}$, with elements equal to 1 indicating the presence of a multilink $\mathbf{m}$ between proteins i and j, and 0 otherwise. We define the multidegree of protein i as:

$$k_i^{\mathbf{m}} = \sum_{j=1}^{N} A_{ij}^{\mathbf{m}} \tag{2}$$

Here, N represents the total number of proteins.

A unipartite duplex network ensemble encompasses all duplexes that adhere to specific constraints, such as the expected multidegree sequences defined in Equation (S2). The probability of observing a unipartite duplex network, denoted as $P(\vec{G})$, is determined using entropy maximization with multidegree constraints $\{k_i^{(1,0)}\}$ and $\{k_i^{(0,1)}\}$, along with their corresponding Lagrangian multipliers $\{\lambda_i^{(1,0)}\}$ and $\{\lambda_i^{(0,1)}\}$, as outlined in [15, 22]. The probability $P(\vec{G})$ factorizes as follows:

$$P(\vec{G}) = \frac{1}{Z} \prod_{ij} \exp\left[- \sum_{\mathbf{m} \neq (0,0),(1,1)} (\lambda_i^{\mathbf{m}} + \lambda_i^{\mathbf{m}}) A_{ij}^{\mathbf{m}} \right] \tag{3}$$

Where:

$$Z = \prod_{ij} \left[1 + \sum_{\mathbf{m} \neq (0,0),(1,1)} e^{-(\lambda_i^{\mathbf{m}} + \lambda_j^{\mathbf{m}})} \right] \tag{4}$$

The multilink probabilities $p_{ij}^{\mathbf{m}}$ are determined by the derivatives of the logarithm of Z with respect to $(\lambda_i^{\mathbf{m}} + \lambda_j^{\mathbf{m}})$. For instance, the probability of observing a positive annotation is expressed as:

$$p_{ij}^{(1,0)} = \frac{e^{-(\lambda_i^{(1,0)}+\lambda_j^{(1,0)})}}{1+e^{-(\lambda_i^{(1,0)}+\lambda_j^{(1,0)})}+e^{-(\lambda_i^{(0,1)}+\lambda_j^{(0,1)})}} \tag{5}$$

Similarly, the probability of observing a negative annotation is given by:

$$p_{ij}^{(0,1)} = \frac{e^{-(\lambda_i^{(0,1)}+\lambda_j^{(0,1)})}}{1+e^{-(\lambda_i^{(1,0)}+\lambda_j^{(1,0)})}+e^{-(\lambda_i^{(0,1)}+\lambda_j^{(0,1)})}} \tag{6}$$

It is important to note that $p_{ij}^{(1,0)} + p_{ij}^{(0,1)} + p_{ij}^{(0,0)}$ equals 1, indicating a normalization of probabilities.

In this theoretical framework, binding prediction is inherently conditional, focusing on the presence of positive and negative annotations for proteins i and j. Therefore, $p_{ij}^{(1,0)}$ and $p_{ij}^{(0,1)}$ are normalized by the probability of observing a generic annotation, i.e., $p_{ij}^{(1,0)} + p_{ij}^{(0,1)}$. For unseen edges, binding prediction is determined by:

$$p_{ij}^{\text{conditional}} = \frac{p_{ij}^{(1,0)}}{p_{ij}^{(1,0)} + p_{ij}^{(0,1)}} \tag{7}$$

In the case of an unseen protein j^*, the binding probability toward a known compound i is calculated as follows:

$$p_{ij^*}^{\text{conditional}} = \frac{\langle p_{ij}^{(1,0)} \rangle_j}{\langle p_{ij}^{(1,0)} \rangle_j + \langle p_{ij}^{(0,1)} \rangle_j} \tag{8}$$

Where $\langle \cdot \rangle_j$ represents the average over all known proteins.

For unseen proteins i^* and j^*, the binding probability is determined based on the overall number of positive ($L^{(1,0)}$) and negative ($L^{(0,1)}$) annotations:

$$p_{i^*j^*}^{\text{conditional}} = \frac{\langle p_{ij}^{(1,0)} \rangle_{ij}}{\langle p_{ij}^{(1,0)} \rangle_{ij} + \langle p_{ij}^{(0,1)} \rangle_{ij}} = \frac{L^{(1,0)}}{L^{(1,0)} + L^{(0,1)}} \tag{9}$$

Here, $\langle \cdot \rangle_{ij}$ indicates the average over all known protein pairs.

4 Annotation Imbalance

The absence of negative samples in PPI databases results in a node-wise class imbalance, where only positive examples are available for the majority of proteins during training. This limitation leads to overpredictions of interactions for these proteins, as the ML models lack exposure to both positive and negative examples [6]. Annotation imbalance is a pervasive issue in interaction databases, as discussed in the context of drug-target binding databases in AI-Bind [6].

To address the annotation imbalance in protein-protein interactions, we utilize TPPNI. As depicted in Figure S4A, TPPNI introduces a significant number of negative samples (3,063,605) compared to subcellular compartmental negatives (738,551) and Negatome (2,211). This approach effectively tackles the challenge of annotation imbalance by providing both positive and negative samples for the majority of the proteins in the training, validation, and test sets of UPNA-PPI (see Figure S4B).

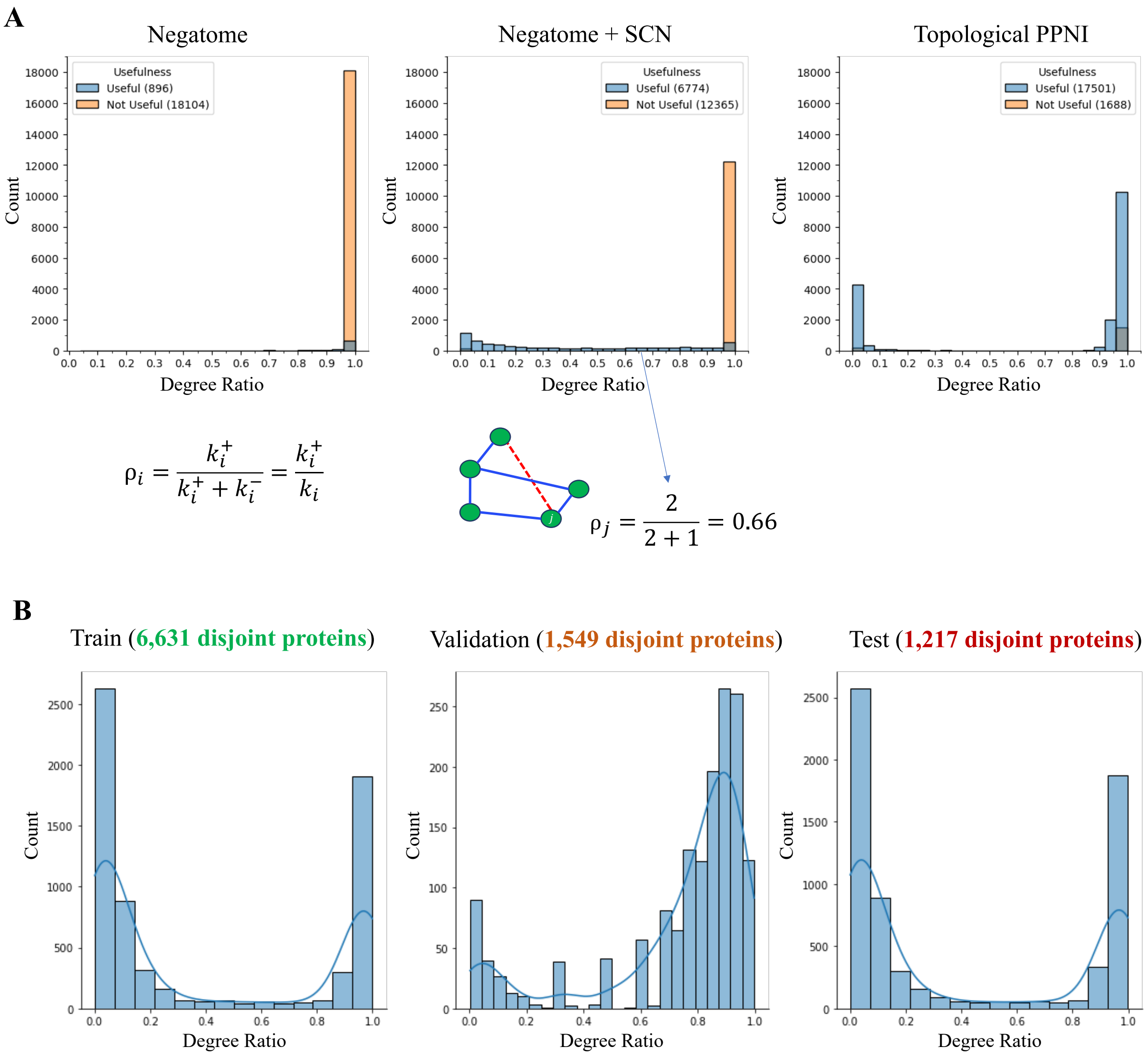

Figure S4: **Removing annotation imbalance and topological shortcuts.** **(A)** Combining the PPI databases and Negatome with only 2k PPNI samples creates a large annotation imbalance in the training data. The majority of proteins only have positive samples and lack negative examples. This imbalance is quantified by degree ratio ($\rho = \frac{k^+}{k}$) [6], which is measured by the ratio of positive annotations (k^+) to total annotations ($k = k^+ + k^-$) associated with a protein. Subcellular Compartmental Negatives (SCN) introduces 778k PPNI examples, rectifying annotation imbalance partially. Finally, TPPNI fixes the issue of annotation imbalance by introducing a substantial number (3 million) of non-interactions. We then filter the interactions and non-interactions involving the proteins having both positive and negative samples for training and testing of UPNA-PPI. Removing proteins with only positive or negative samples helps UPNA-PPI circumvent learning shortcuts related to predicting that some proteins always interact or do not interact. **(B)** Using only TPPNI we remove annotation imbalance from all train, validation, and test data of UPNA-PPI. Here, we visualize the degree ratio distributions in train, validation, and test datasets for the first fold.

5 Fundamental Nature of L3 paths

L = 3 is the fundamental unit for odd path lengths in complementarity-driven networks. In Figure S5A, we show that an L = 5 path resolves into two L = 3 paths. Similarly, an L = 7 path would resolve into three L = 3 paths, and so on.

Furthermore, Figure S5B shows the connection probability for 1,000 protein pairs ranked by different powers of the adjacency matrix of a PPI network, counting all paths of length L = 2, 3, . . . , 8. L = 3 paths are the most informative on direct connectivity [23]. Thus, L = 3 paths are the most informative of protein-protein interactions.

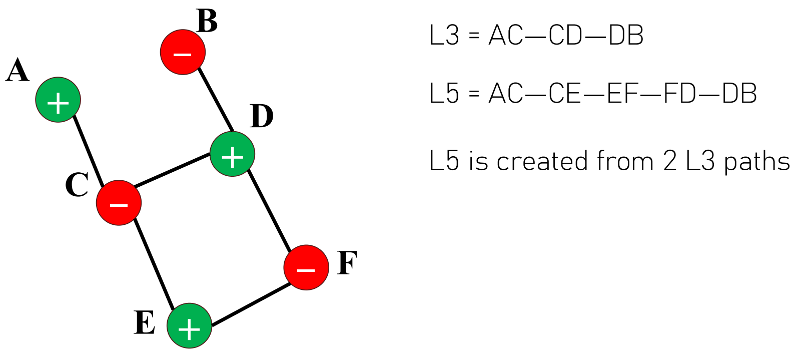

Figure S5: **Fundamental Nature of L3 paths in Complimentarily-driven Networks. (A)** L=5 path resolves into two L=3 paths in a charge-complementarity-driven network setting. Similarly, L=7 paths resolve into 3 stacked L=3 paths. Hence, L=3 paths form the fundamental cycles in complementarity-driven networks. L=3 paths are more informative of the connection probabilities in PPI networks as demonstrated by Kovács et al. in [23], they present the connection probability for various path lengths ranging from $L = 2$ to $L = 8$.

6 Degree-Preserved Edge-Swap in PPIs

We randomly swapped 50% of the edges in train and validation data keeping the degrees of the nodes unchanged. It means that 25% of the edges produced other positive edges and the other 25% were potential negatives. However, we observe in Figure 6D that UPNA-PPI shows high robustness for link deletion. The inductive test performance slightly drops or remains unchanged when 20% of the training edges are removed at random. Thus, we observe the test performance not being substantially affected by the degree-preserved edge swap.

Furthermore, we looked into the relationship between the prediction of UPNA-PPI and the degrees of the end nodes of a PPI edge. We know that the configuration model (Chung-Lu model) leverages topological shortcuts for link prediction [24]. Hence, in the presence of topological shortcuts, the product of the degrees of the end nodes of a PPI edge should highly correlate with the model predictions. However, this is not the case for UPNA-PPI. We plot in Figure S6, the product of the degrees of the end nodes of PPI edges in the first fold of the test set with UPNA-PPI prediction, and observe a Pearson's correlation coefficient of 0.2 (low correlation). This confirms that the UPNA-PPI predictions (inductive setting) are devoid of degree-based topological shortcuts.

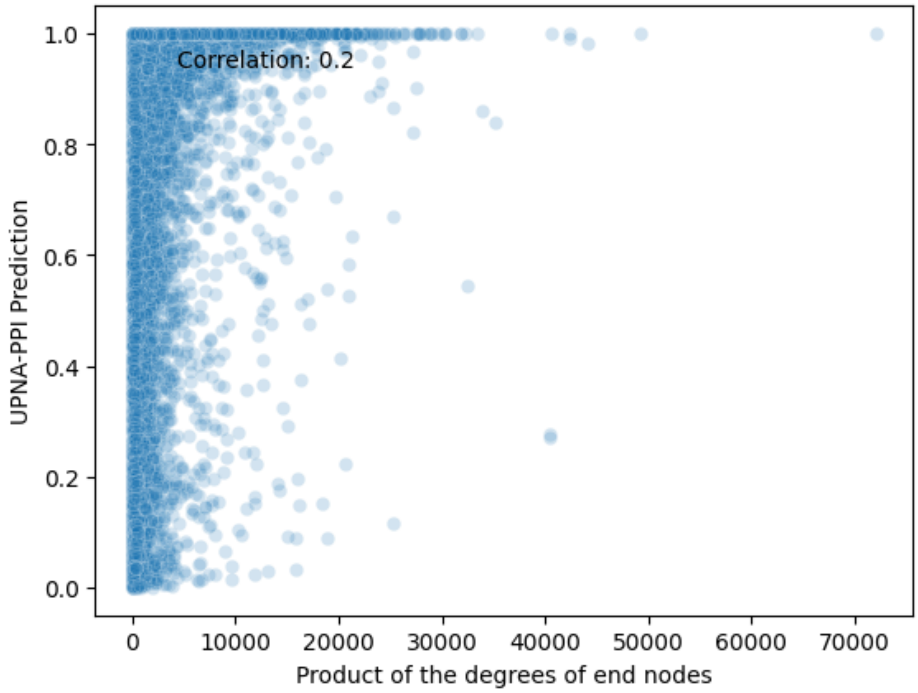

Figure S6: **UPNA-PPI Predictions and Node-Degrees.** In a traditional configuration model, the probability of a link between two nodes is proportional to the product of the degrees of the end nodes of the link. Here, we plot the product of the degrees of the end nodes of the edges in UPNA-PPI test set of the first fold. We observe low correlation between the UPNA-PPI prediction (inductive setting) and the degree products. Hence, we conclude that UPNA-PPI is devoid of degree-based topological shortcuts.

7 Coefficient of Variation for GPCR Predictions.

To further investigate UPNA-PPI output, we looked into the coefficient of variation (for the 5-folds) as illustrated in Figure S7. Coefficient of variation is able to capture the combined effect of the standard deviation and mean of the predictions from 5-folds of UPNA-PPI.

8 Alternate Protein Embeddings

We evaluate UPNA-PPI against another alternative protein representation learning methodology. Variational autoencoders (VAE) [25] are utilized to derive protein embeddings from amino acid sequences by minimizing both reconstruction loss and distributional loss. We implement VAE-based embeddings following the methodology proposed by Hawkins-Hooker et al. [26]. Initially, we generate one-hot encodings for the amino acid residues and employ these encodings to represent the sequences. Subsequently, a VAE is trained to produce 100-dimensional representations of the sequences. Alternatively, we employ a message-passing neural network (MPNN) [27] on 3D protein structures sourced from the Protein Data Bank (PDB) and those predicted by AlphaFold [28]. However, our examination reveals that Protvec outperforms both VAE and MPNN in inductive testing scenarios (refer to Table S2). Furthermore, we create a simple embedding of the 3D protein structures from the PDB files. We one hot encode the atoms and flatten out the information in the PDB files (atoms, coordinates, and electron den-

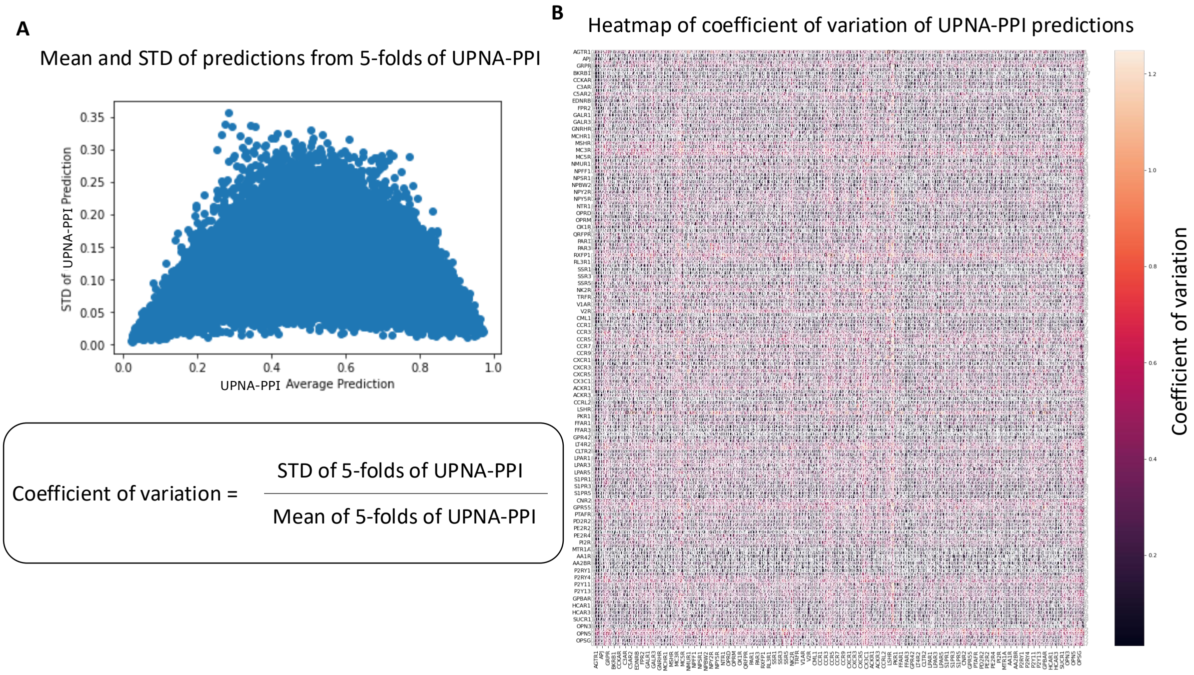

Figure S7: **Coefficient of variation.** **(A)** We plot the standard deviation (STD) vs. mean of the predictions from the 5-folds of UPNA-PPI for GPCR proteins. We observe that the STD is lower for the predictions near 0 and 1, indicating that UPNA-PPI models from 5-folds agree upon the top and the bottom predictions. Whereas, the predictions in between have higher STD. Thus, we define coefficient of variation of UPNA-PPI predictions as the ratio of the STD and mean from the 5-folds. Lower value of this coefficient suggests lower STD with high average, this high confidence interaction predictions. **(B)** We plot the heatmap of the coefficient of variation from UPNA-PPI predictions for 170 GPCR proteins. The darker dots correspond to the high confidence interaction predictions.

sities). We use zero-padding at the end for making the embeddings of same length for different proteins. We observe that these simpler 3D embeddings, although performs poorly compared to Protvec, show improved performance compared to the MPNN approach.

Table S2: **Comparing Different Protein Embeddings Methods in Inductive Tests.** We compare Protvec-based protein representations with VAE and MPNN-based approaches. In both cases, Protvec performs better. Interestingly, a simpler one-hot encoding-based approach on the PDB files provides better performance compared to the MPNN-based protein embeddings.

Model	AUROC	AUPRC	Hits@Top1000	Hits@Top10,000
Protvec	0.79 ± 0.007	0.87 ± 0.009	0.92 ± 0.015	0.87 ± 0.010
VAE	0.64 ± 0.05	0.63 ± 0.05	0.74 ± 0.13	0.64 ± 0.05
MPNN	0.59 ± 0.06	0.62 ± 0.07	0.25 ± 0.008	0.024 ± 0.001
PDB	0.74 ± 0.03	0.72 ± 0.03	0.68 ± 0.01	0.55 ± 0.02

9 Sequence Identities Between the Train and Test Sets

To obtain the sequence identities we used BLASTp (version 2.9.0) with default settings [29]. Next we randomly sampled 1000 proteins from train and test datasets for each of the five folds, and ran BLASTp for all pairs of proteins between the train and test datasets. The reason for sampling is the number of test-train protein pairs becomes large to run BLASTp, moreover 1,000 samples creates 1,000 * 1,000 = 1,000,000 train-test protein pairs, which is a sufficiently large sample size for investigating sequence identities between the train and test sets. Lastly, this sampling scenario is conducted on each of the five folds, providing five independent datasets of 1,000,000 train-test protein pairs.

We observe a similar trend within all 5 folds as demonstrated in Figure S8. BLASTp reports the percentage of identity calculated as ("Number of Identical Matches" / "Length of the Alignment") $*$ 100 for each train-test protein pair, and it is likely that a pair could have multiple alignments in different regions, hence we report the average percentage of identity for each pair. We observe a significantly low percentage of identity between train and test datasets (average percentage of identity=35.6% with average std=3.57%), especially for most pairs BLASTp did not find any alignment, indicating no similarity (0 identity).

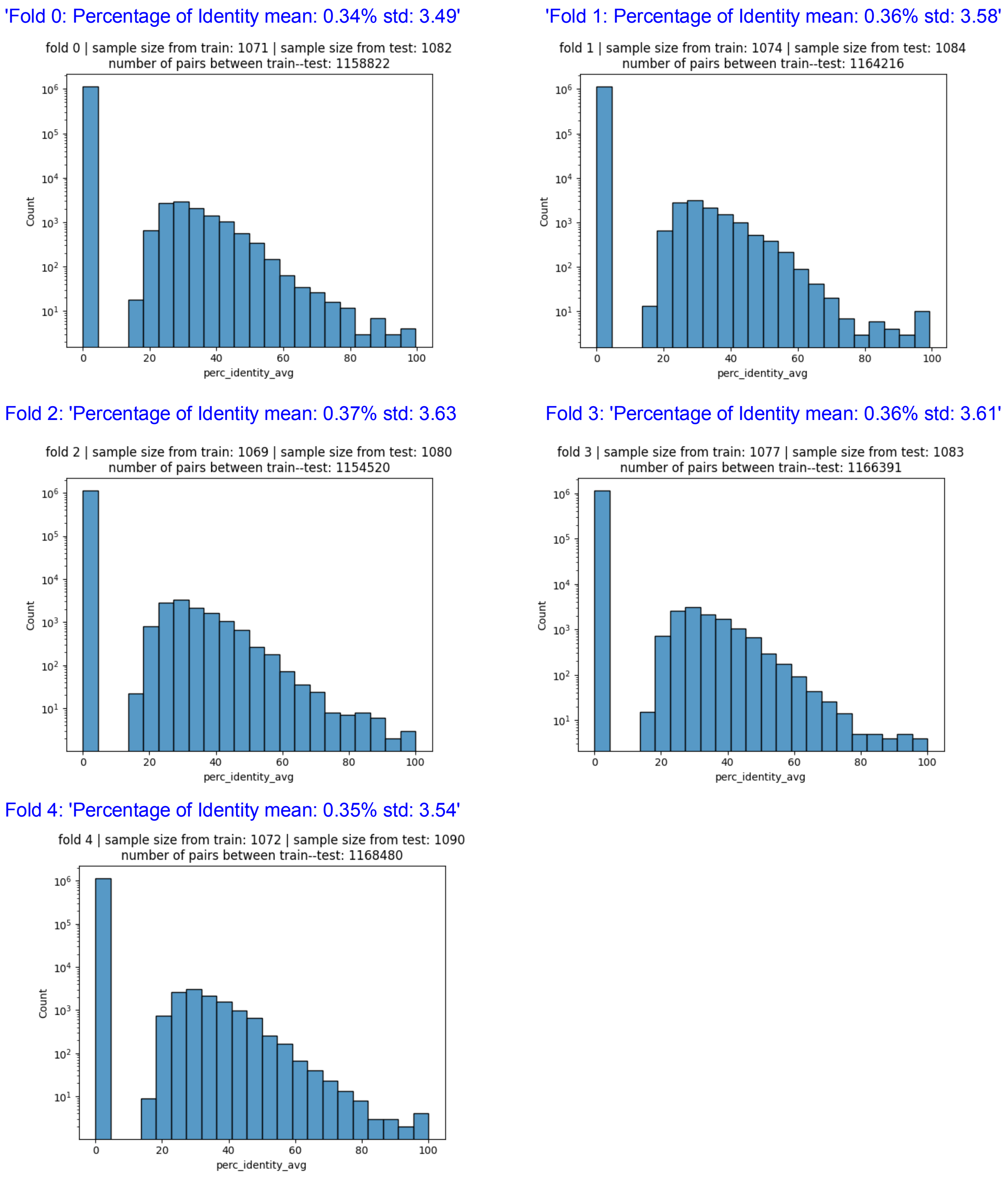

Figure S8: **Sequence Identities Between the Train and Test Sets.** We used BLASTp (with default parameters) to investigate if there is a high identity (similarity) between train and test protein sequences, which could cause shortcut learning and overfitting. We observe that our inductive train-test folds obtained by leveraging protein-protein interactions (network properties), indeed offer dissimilar train-test protein pairs that are ideal for inductive test and enhance the generalizability of UPNA-PPI.

10 UPNA Capturing PPI Network Properties

In this section, we demonstrate that UPNA-PPI predictions can capture first and second-order network properties of PPI networks unseen during training. The analysis examines the structural similarities between the original network, $G_{Original}$, and the predicted network,

$G_{Predicted}$, within the protein-protein interaction (PPI) subgraph constructed from the test dataset of Fold 1 in UPNA-PPI training. The network $G_{Predicted}$ represents the links predicted in an inductive setting by the trained UPNA-PPI model for Fold 1, using a threshold of 0.5 to assign links (see Figure 9a, such that a protein-protein pair is included in $G_{Predicted}$ if the predicted probability is ≥ 0.5. The results demonstrate that the predicted links closely replicate key structural properties of $G_{Original}$, including the degree distribution (see Figure 9b) and the node-wise triangle distribution (see Figure 9c). Furthermore, $G_{Predicted}$ exhibits a similar relationship between the node-wise triangle count and degree as observed in $G_{Original}$ (see Figure 9d), with Pearson's correlation coefficients of 0.899 for $G_{Original}$ and 0.894 for $G_{Predicted}$.

Furthermore, we test the network reconstruction capabilities of UPNA-PPI with random negative samples. G_{random} is constructed by predicting links in an inductive setting but with random negative sampling from G_{train} instead of the derived TPPNI. Using a threshold of 0.5, links in G_{random} are identified such that a protein-protein pair with a predicted probability ≥ 0.5 is assigned a link in $G_{Predicted}$ (see Figure 10a). The distribution of predicted probabilities in G_{random} shifts toward lower values compared to the TPPNI-based predictions. The degree distribution of $G_{Predicted}$ closely matches that of $G_{Original}$, whereas G_{random} exhibits more divergence (see Figure 10b). Node-wise triangle distributions between $G_{Original}$ and $G_{Predicted}$ are more similar than those between $G_{Original}$ and G_{random} (see Figure 10c). Both $G_{Predicted}$ and G_{random} replicate the relationship between node-wise triangle counts and degrees observed in $G_{Original}$, with Pearson's correlation coefficients of 0.899, 0.894, and 0.9073 for $G_{Original}$, $G_{Predicted}$, and G_{random}, respectively (see Figure 10d). Finally, while G_{random} closely approximates node-wise L3 counts (4-cycles) from $G_{Original}$, it significantly underestimates the number of L3 paths (see Figure 10e,f).

In Figure 10a, we observe that UPNA-predicted degree density for the low-degree nodes is higher. Since topological shortcuts are circumvented in inductive tests, fewer links are predicted by UPNA for the hub nodes. Further investigation could shed light on the characteristics of links predicted by UPNA for hubs, particularly whether UPNA captures higher-confidence links, while topological shortcuts predict both high- and low-confidence edges.

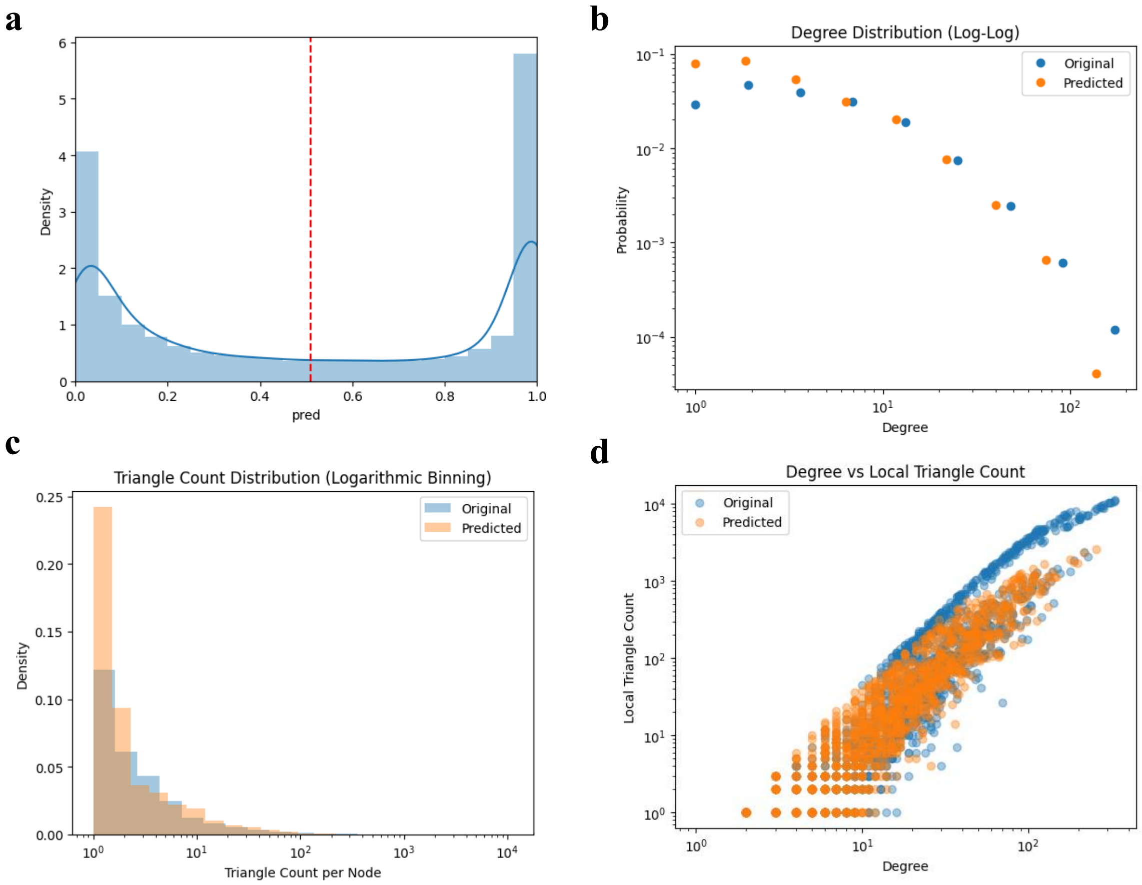

Figure S9: **UPNA Capturing PPI Network Properties.** Here, the original network $G_{Original}$ refers to the PPI subgraph constructed by the test dataset of Fold 1 of UPNA-PPI training. The predicted graph $G_{Predicted}$ refers to the graph constructed by the links predicted in an inductive setting by the trained model in Fold 1. **(a)** We use a threshold of 0.5 to identify the links of $G_{Predicted}$, i.e., is the UPNA-PPI predicted probability is ≥ 0.5 that protein-protein pair is assigned a link in $G_{Predicted}$. **(b)** We observe that UPNA-PPI predicted links closely capture the degree distribution of $G_{Original}$. **(c)** UPNA-PPI predicted links closely capture the node-wise triangle distribution of $G_{Original}$. **(d)** $G_{Predicted}$ shows a similar node-wise triangle count vs degree relationship as $G_{Original}$ with the Pearson's correlation coefficients being 0.899 and 0.894 for $G_{Original}$ and $G_{Predicted}$, respectively.

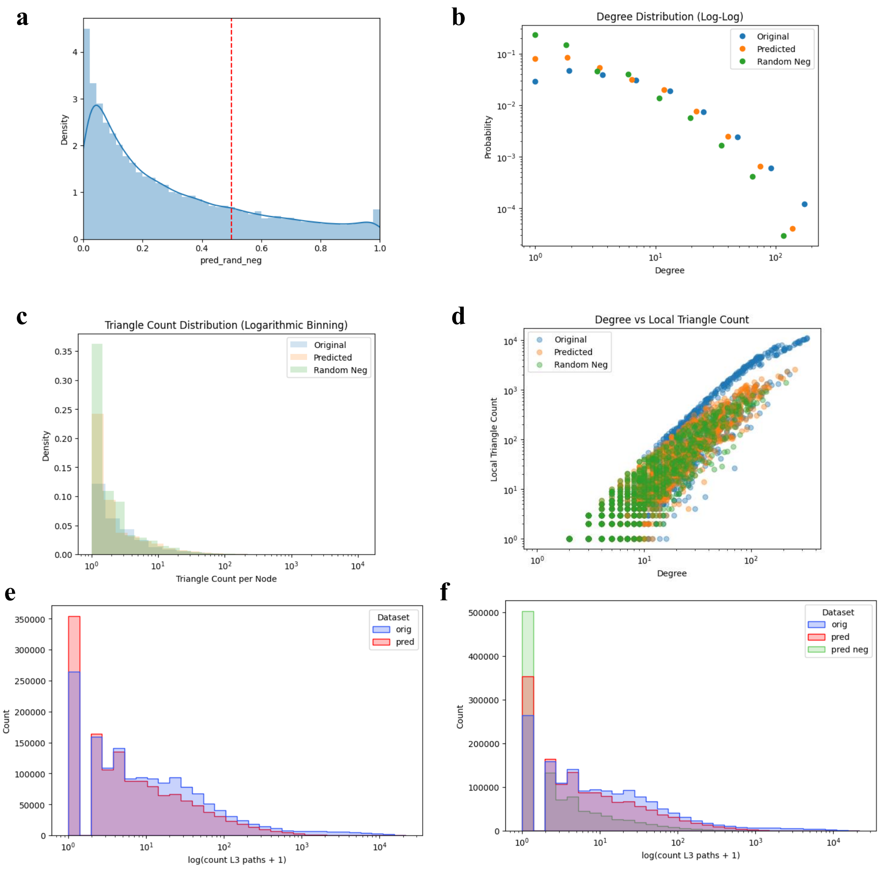

Figure S10: **UPNA with random negative sampling in capturing PPI Network Properties.** Here, the original network $G_{Original}$ refers to the PPI subgraph constructed by the test dataset of Fold 1 of UPNA-PPI training. The predicted graph $G_{Predicted}$ refers to the graph constructed by the links predicted in an inductive setting by the trained model in Fold 1. Furthermore, I construct G_{random} by predicting links in an inductive fashion by training the same model using random negative sampling from G_{train} instead of the derived TPPNI. **(a)** We use a threshold of 0.5 to identify the links of G_{random}, i.e., is the UPNA-PPI predicted probability is ≥ 0.5 that protein-protein pair is assigned a link in $G_{Predicted}$. We observe that the distribution of the predicted probabilities have shifted towards lower values when random negative samples are used compared to TPPNI. **(b)** We observe that $G_{Original}$ closely capture the degree distribution of $G_{Original}$. The degree distribution for G_{random} is more divergent compared to that of $G_{Original}$. **(c)** Node-wise triangle distributions are more similar between $G_{Original}$ and $G_{Predicted}$, compared to $G_{Original}$ and G_{random}. **(d)** Both $G_{Predicted}$ and G_{random} show similar node-wise triangle count vs degree relationship as $G_{Original}$ with the Pearson's correlation coefficients being 0.899, 0.894, and 0.9073 for $G_{Original}$, $G_{Predicted}$, and G_{random}, respectively. **(e)-(f)** G_{random} closely replicates the node-wise L3 counts (4-cycles) from $G_{Original}$, whereas G_{random} produces significantly less number of L3 paths.

References

[1] Jaccard, P. THE DISTRIBUTION OF THE FLORA IN THE ALPINE ZONE.1. *New Phytologist* **11**, 37–50 (1912). URL https://doi.org/10.1111/j.1469-8137.1912.tb05611.x.

[2] Qi, Y., Bar-Joseph, Z. & Klein-Seetharaman, J. Evaluation of different biological data and computational classification methods for use in protein interaction prediction. *Proteins: Structure, Function, and Bioinformatics* **63**, 490–500 (2006). URL https://doi.org/10.1002/prot.20865.

[3] Feng, Y., Wang, Q. & Wang, T. Drug target protein-protein interaction networks: A systematic perspective. *BioMed Research International* **2017**, 1–13 (2017). URL https://doi.org/10.1155/2017/1289259.

[4] Barabási, A.-L. & Albert, R. Emergence of scaling in random networks. *Science* **286**, 509–512 (1999). URL https://doi.org/10.1126/science.286.5439.509.

[5] Tan, J., Yang, J., Wu, S., Chen, G. & Zhao, J. A critical look at the current train/test split in machine learning (2021). 2106.04525.

[6] Chatterjee, A. *et al.* Improving the generalizability of protein-ligand binding predictions with AI-bind. *Nature Communications* **14** (2023). URL https://doi.org/10.1038/s41467-023-37572-z.

[7] Chatterjee, A., Walters, R., Menichetti, G. & Eliassi-Rad, T. Disentangling node attributes from graph topology for improved generalizability in link prediction (2023). URL https://doi.org/10.48550/arXiv.2307.08877.

[8] ERDdS, P. & R&wi, A. On random graphs i. *Publ. math. debrecen* **6**, 18 (1959).

[9] Piñero, J. *et al.* The DisGeNET knowledge platform for disease genomics: 2019 update. *Nucleic Acids Research* (2019). URL https://doi.org/10.1093/nar/gkz1021.

[10] Genes, G. Rare diseases, common challenges. *Nature Genetics* **54**, 215–215 (2022). URL https://doi.org/10.1038/s41588-022-01037-8.

[11] Huang, K. *et al.* DeepPurpose: a deep learning library for drug–target interaction prediction. *Bioinformatics* (2020). URL https://doi.org/10.1093/bioinformatics/btaa1005.

[12] Qiagen biomedical knowledge base (2023.2).

[13] Hu, F., Jiang, J., Wang, D., Zhu, M. & Yin, P. Multi-PLI: interpretable multi-task deep learning model for unifying protein–ligand interaction datasets. *Journal of Cheminformatics* **13** (2021). URL https://doi.org/10.1186/s13321-021-00510-6.

[14] Jha, K., Saha, S. & Singh, H. Prediction of protein–protein interaction using graph neural networks. *Scientific Reports* **12**, 8360 (2022).

[15] Menichetti, G. & Remondini, D. Entropy of a network ensemble: Definitions and applications to genomic data. *Theoretical Biology Forum* **107**, 77–87 (2014).

[16] Teru, K. K., Denis, E. & Hamilton, W. L. Inductive relation prediction by subgraph reasoning (2019). URL https://arxiv.org/abs/1911.06962.

[17] Park, Y. & Marcotte, E. M. Flaws in evaluation schemes for pair-input computational predictions. *Nature Methods* **9**, 1134–1136 (2012). URL https://doi.org/10.1038/nmeth.2259.

[18] Chatterjee, A., Walters, R., Menichetti, G. & Eliassi-Rad, T. Inductive link prediction in static and temporal graphs for isolated nodes. In *Temporal Graph Learning Workshop @ NeurIPS 2023* (2023). URL https://openreview.net/forum?id=DRrSYKNhD1.

[19] Meng, R. *et al.* Attention diversification for domain generalization (2022). 2210.04206.

[20] Bardes, A., Ponce, J. & LeCun, Y. VICReg: Variance-invariance-covariance regularization for self-supervised learning. In *International Conference on Learning Representations* (2022). URL https://openreview.net/forum?id=xm6YD62D1Ub.

[21] Alberts, B. & etc. (eds.) *Molecular biology of the cell* (CRC Press, Boca Raton, FL, 2002), 4 edn.

[22] Menichetti, G. & Remondini, D. Entropy of a network ensemble: definitions and applications to genomic data. *Theor Biol Forum* **107**, 77–87 (2014).

[23] Kovács, I. A. *et al.* Network-based prediction of protein interactions. *Nature Communications* **10** (2019). URL https://doi.org/10.1038/s41467-019-09177-y.

[24] Chung, F. & Lu, L. The average distances in random graphs with given expected degrees. *Proceedings of the National Academy of Sciences* **99**, 15879–15882 (2002).

[25] Kingma, D. P. & Welling, M. Auto-encoding variational bayes (2022). 1312.6114.

[26] Hawkins-Hooker, A. *et al.* Generating functional protein variants with variational autoencoders. *PLOS Computational Biology* **17**, e1008736 (2021). URL http://dx.doi.org/10.1371/journal.pcbi.1008736.

[27] Gilmer, J., Schoenholz, S. S., Riley, P. F., Vinyals, O. & Dahl, G. E. Neural message passing for quantum chemistry. In *Proceedings of the 34th International Conference on Machine Learning - Volume 70*, ICML'17, 1263–1272 (JMLR.org, 2017).

[28] Wang, L., Liu, Y., Lin, Y., Liu, H. & Ji, S. ComENet: Towards complete and efficient message passing for 3d molecular graphs. In Oh, A. H., Agarwal, A., Belgrave, D. & Cho, K. (eds.) *Advances in Neural Information Processing Systems* (2022). URL https://openreview.net/forum?id=mCzMqeWSFJ.

[29] Camacho, C. *et al.* Blast+: architecture and applications. *BMC bioinformatics* **10**, 1–9 (2009).
